# OPIOID-EXPRESSING B CELLS SILENCE TUMOR-INFILTRATING NOCICEPTOR NEURONS

**DOI:** 10.21203/rs.3.rs-7389517/v1

**Published:** 2025-09-03

**Authors:** Tuany Eichwald, Maryam Ahmadi, Andre Martel Matos, Amin Reza Nikpoor, Karine Roversi, Mohammad Balood, Fendi Obuekwe, Marci L. Nilsen, Ayana T. Ruffin, Gabryella Pinheiro, Laura Brabenec, Moutih Rafei, Paola Vermeer, Tullia C. Bruno, Nicole N Scheff, Sebastien Talbot

**Affiliations:** 1 Department of Biomedical and Molecular Sciences, Queen’s University. Kingston, Canada; 2 Department of Physiology and Pharmacology, Karolinska Institutet. Solna, Sweden.; 3 Department of Pharmacology and Physiology, University de Montreal. Montreal, Canada; 4 Department of Neurobiologyand Hillman Cancer Center, University of Pittsburgh. Pittsburgh, USA; 5 Cancer Biology and Immunotherapies Group, Sanford Research. Sioux Falls, USA

**Keywords:** Cancer Neuroscience, Neuro-Immunology, B Cells, Melanoma, Pain, Nociceptin, Head and Neck Cancer

## Abstract

Nociceptor neurons, which transmit pain signals, also regulate immunity by releasing immunomodulatory neuropeptides. In head and neck squamous cell carcinoma (HNSCC) and melanoma, our research has shown that tumor-innervating nociceptors modulate anti-tumor immunity through the release of calcitonin gene-related peptide (CGRP) and its interaction with receptor activity-modifying protein 1 (RAMP1). A retrospective analysis of clinical charts from HNSCC patients revealed that higher pain levels correlated with increased opioid use, perineural invasion, and decreased B-cell infiltration—factors associated with poorer survival outcomes. *In silico* single-cell RNA sequencing demonstrated that opioid use in HNSCC patients downregulates nociceptin/orphanin FQ (N/OFQ), an endogenous ligand for opioid receptor-like-1 (OPRL1). We identified B cells as the primary source of N/OFQ and observed that high expression of either *Pnoc* or *Oprl1* correlates with better survival in both melanoma and HNSCC. In a mouse model of oral squamous cell carcinoma (oSCC), we found that nociceptor neurons in tongue tumors overexpress *Oprl1* and exhibit severe mechanical pain hypersensitivity. Compared to healthy tissue, oSCC tumors have dense infiltration of nociceptor fibers and N/OFQ-expressing B cells. Pharmacological blockade of *Oprl1* reduced HNSCC-induced mechanical pain. In a melanoma mouse model, tumor-innervating neurons also overexpressed *Oprl1*, and similar overexpression was observed when DRG neurons were co-cultured with B16F10 cells. Activating OPRL1 reduced tumor size, enhanced cytotoxic T-cell infiltration, and relieved cancer-induced thermal hypersensitivity. In contrast, depleting CD19^+^ B cells or blocking OPRL1 led to increased tumor growth, reduced CD8^+^ T-cell infiltration and cytotoxic potential, exacerbated pain, and elevated CGRP levels. Moreover, we discovered that Ramp1^+^ B cells express *Pnoc*, but this expression is suppressed by CGRP. Blocking RAMP1 reduced tumor growth and promoted B-cell *Pnoc* expression. Overall, these findings suggest that targeting the N/OFQ and RAMP1 pathways could bolster anti-tumor immunity while simultaneously alleviating cancer-induced pain.

## INTRODUCTION.

Solid tumors often develop new peripheral nerve fibers originating from both autonomic and sensory nervous systems. Recent findings show that nociceptive neurons infiltrating tumors can modulate cancer immunosurveillance via calcitonin gene-related peptide (CGRP). In the tumor microenvironment, CGRP triggers immune exhaustion by stimulating receptor activity-modifying protein 1 (RAMP1) on CD8 T-cells^1–4^. However, selectively denervating these nerves is not a viable clinical option: it requires early intervention and can cause severe organ dysfunction by disrupting sensory feedback. New strategies are urgently needed to modulate peripheral neuronal activity within the tumor microenvironment (TME) without compromising overall function and survivorship.

Currently, exogenous opiates are the standard treatment for cancer-related pain; the primary mechanism of action is in the spinal cord and brain through binding to the widely expressed μ-opioid receptor (OPRM1), with minimal direct inhibition of the primary sensory nerves innervating the tissue^5^. Unfortunately, opiates can produce significant side effects, have limited efficacy, pose a high risk of addiction, and cause immunosuppression^6,7^. In contrast, the endogenous opioid system—primarily limited to beta-endorphin, met-enkephalin, and leu-enkephalin—remains underexplored for its potential to regulate neuronal activity and bolster anti-tumor immunity^8^. Preclinical head and neck and pancreatic cancer models reveal that the endogenous opioid system influences neuronal activity and pain^9,10^. Specifically, beta-endorphin released by neutrophils can alleviate cancer-related pain in an orthotopic oral cancer model^9^; however, neutrophil infiltration into tumors can promote tumor growth, metastasis, and resistance to therapy. While these data provide premise for an immune-mediated analgesic mechanism, alternative sources for endogenous opioid signaling should be explored.

Nociceptin/Orphanin FQ (N/OFQ) is a 17-amino acid neuropeptide that serves as the endogenous ligand for the opioid receptor-like 1 (OPRL1) receptor^11,12^. Despite belonging to the opioid family, N/OFQ has distinct functional properties compared to traditional opioids^13–15^. Mice lacking the N/OFQ gene (*Pnoc*^−/−^) experience heightened pain sensitivity in response to formalin-induced inflammation^16,17^. N/OFQ can also selectively and dose-dependently reduce cancer-induced bone pain^18^. Further studies indicate that N/OFQ signaling can influence tumor growth, metastasis, angiogenesis, cancer-related pain, and immune responses, with effects that can be either anti-tumorigenic or pro-tumorigenic depending on the tumor type and environment^19–21^.

Tumor-infiltrating B cells are an important source of N/OFQ within the TME^22^, and they are increasingly recognized as key contributors to T cell-mediated anti-tumor responses. Clinical data suggest that the presence of B cells in or near tumors correlates with better prognosis and improved responses to immunotherapy^23^. Although activated B cells often signal a stronger T cell response^23^, their direct effect on treatment outcomes remains unclear. More research is needed to elucidate the interactions between B cells and other cells in the TME, which could reveal avenues to enhance therapeutic efficacy.

In this study, we propose that endogenous, immune-mediated N/OFQ signaling in the TME suppresses nociceptor neuron activity through the OPRL1, thereby boosting anti-tumor immunity. We will investigate the mechanistic roles of N/OFQ and OPRL1 in head and neck squamous cell carcinoma (HNSCC) and melanoma patients and translate these findings into a mouse model. We will also determine how N/OFQ:OPRL1 signaling affects tumor progression and immune responses in murine melanoma models. Our goal is to identify new therapeutic targets for cancer patients, including exploiting opioid-producing B cells as novel modulators of cancer pain and progression.

## RESULTS.

### N/OFQ and OPRL1 Expression in Cancer

Melanoma skin cancer and head and neck tumors are densely innervated by nociceptor neurons. In both malignancies, locally released sensory neurotransmitter, calcitonin gene related peptide (**CGRP**) contributes to impaired anti-tumor immunity and T-cell exhaustion. Our data suggest that tumor-induced pain precedes T-cell exhaustion in these tumors. However, the precise source of this pain and its implications for patient survival remain unclear. To investigate these questions, we focused on Nociceptin/Orphanin FQ (**N/OFQ**) and its receptor (**OPRL1**) to better understand their roles in tumor-associated pain.

**Fig. 1.**
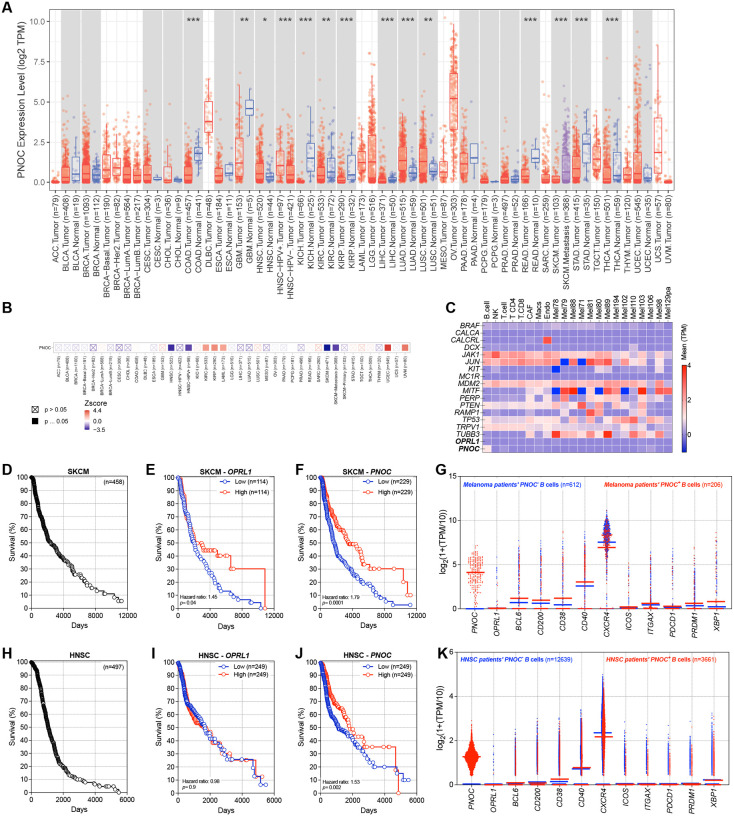
*OPRL1* and *PNOC* impact survival in patients with skin cutaneous melanoma and head and neck squamous cell carcinoma. **A**, *In silico* analysis of *PNOC* expression across cancer datasets^51–53^ revealed elevated *PNOC* levels in several tumor types, including higher *PNOC* expression in head and neck squamous cell carcinoma (HNSC) tumors compared to normal tissue, and in metastatic skin cutaneous melanoma (SKCM) compared to primary SKCM tumors. **B**, *In silico* analysis of The Cancer Genome Atlas (TCGA) data^54^ was used to correlate patient survival with relative *PNOC* expression (primary biopsy bulk RNA sequencing). Cox proportional hazard model show that higher *PNOC expression* levels exhibit prolonged survival in both SKCM and HNSC (positive coefficients; red squares). **C**, *In silico* analysis of single-cell RNA sequencing data of human melanoma (GSE115978)^55^ revealed that intratumoral *PNOC* is exclusively expressed in tumor-infiltrating B-cells. **D–F**, *In silico* analysis of TCGA data^54^ was used to correlate the survival rate in 459 patients with melanoma (**D**) with the relative expression of *OPRL1* (**E**) and *PNOC* (**F**) (primary biopsy bulk RNA sequencing). Higher expression of either *OPRL1* or *PNOC* correlate with improved survival compared to patients with low expression of both genes. **G**, *In silico* analysis of single-cell RNA sequencing data of human melanoma (GSE115978)^55^ revealed that intratumoral *PNOC*-expressing B-cells overexpress several anti-tumoral markers—ranging from immunosuppressive molecules (*CD200*) to those involved in antigen presentation (*CD40*) and effector functions (*CD38*)—in comparison to *PNOC*-negative B cells. In addition, *PNOC*-expressing B-cells show higher expression of key transcription factors (*BCL6*, *PRDM1*, *XBP1*) that govern B-cell fate decisions (germinal center vs. plasma cell) and drive the production of high-affinity, tumor-targeting antibodies. Data also indicate that ~33% of melanoma (206 out of 612) infiltrating B-cells express *PNOC*. **H–J**, *In silico* analysis of TCGA data^54^ was used to correlate the survival rate in 497 patients with HNSC (**H**) with the relative expression of *OPRL1* (**I**) and *PNOC* (**J**) (primary biopsy bulk RNA sequencing). Higher expression of *PNOC* correlate with improved survival compared to patients with low *PNOC* expression. *OPRL1* expression was not significantly affect HNSC patient survival (**I**). **K**, *In silico* analysis of single-cell RNA sequencing data of human HNSC (GSE164690)^56^ indicated that intratumoral *PNOC*-expressing B-cells strongly overexpress several anti-tumoral markers—i.e. antigen presentation (*CD40*), effector functions (*CD38*), and key transcription factor (*XBP1)* relative to *PNOC*-negative B-cells. Data also indicate that ~29% of HNSCC (3,661 out of 12,639) infiltrating B-cells express *PNOC*. Data are shown as PNOC Log^2^ TPM expression box-and-whisker plots (extending from the minimum to the maximum values, with the box spanning the 25th to 75th percentiles and the middle line indicating the median) (**A**), as a heatmap showing both Z-scores and significance annotated (**B**), as a heatmap displaying gene expression as log_2_ transcripts per million (TPM) (**C**), as Mantel–Cox regression (**D, E, F, H, I, J**), or as scatter dot plot with medians (**G, K**). n as follows: **A:** 3–1093 per group; **B:** 36–1100; **D:** 458; **E:** 114 per group; **F:** 229 per group; **G:** 612 (Pnoc^neg^) and 206 (Pnoc^pos^); **H:** 497; **I, J:** 249 per group; **K:**12,639 (Pnoc^neg^) and 3,661 (Pnoc^pos^). p values were determined by Wilcoxon test (**A**) Cox proportional hazard model (**B**), or Mantel–Cox regression (**E, F, I, J**).

Analysis of The Cancer Genome Atlas (**TCGA**) revealed significantly higher *PNOC* expression (the gene encoding N/OFQ) in tumors compared with normal tissues (Fig. 1A). Notably, the most pronounced differences in survival were observed in head and neck squamous cell carcinoma (**HNSCC**) and melanoma (**SKCM**; Fig. 1B). Given the strong correlation with patient survival, we next used single-cell RNA sequencing data from patients to determine the cellular source of *PNOC* within tumors.

*In silico* analyses of SKCM (Fig. 1C, SF. 1A–D) and HNSCC (SF. 2A–D) single-cell RNA-seq datasets showed that *PNOC* is predominantly expressed by tumor-infiltrating B-cells, with little to no expression in other immune or tumor cells. Using the ImmGen RNA-seq database, we confirmed that *PNOC* is largely restricted to B-cells in rodents and humans (SF. 1E, SF. 3). Consistent with these findings, when we employed the “FindMarker” function to identify genes co-expressed with *PNOC*, and *CD79A*, a crucial component of the B cell receptor, was the first gene identified in this process (SF. 1E).

Analysis of a separate dataset confirmed that high *PNOC* and *OPRL1* levels are associated with improved survival in SKCM patients (Fig. 1D–F). Further investigation revealed that *PNOC*-expressing B-cells exhibit a predominantly activated phenotype, showing high expression of *BCL6*, *XBP1*, and *CD38* (Fig. 1G). In HNSCC, we found that although elevated *OPRL1* expression did not confer a clear survival benefit, higher *PNOC* levels correlated with more favorable clinical outcomes and lower tumor grade (Fig. 1H–J). In this case, we also uncovered that intratumoral *PNOC*-expressing B-cells strongly overexpress several anti-tumoral markers—particularly those involved in antigen presentation (*CD40*) and effector functions (*CD38, CXCR4*)—relative to *PNOC*-negative B-cells. Moreover, these *PNOC*-expressing B-cells upregulate the key transcription factor *XBP1*, which is essential for B cell fate decisions (germinal center vs. plasma cell) and the generation of high-affinity, tumor-targeting antibodies (Fig. 1K).

### Head and Neck Cancer (**HNC**), Perineural Invasion, and Pain

**Fig. 2.**
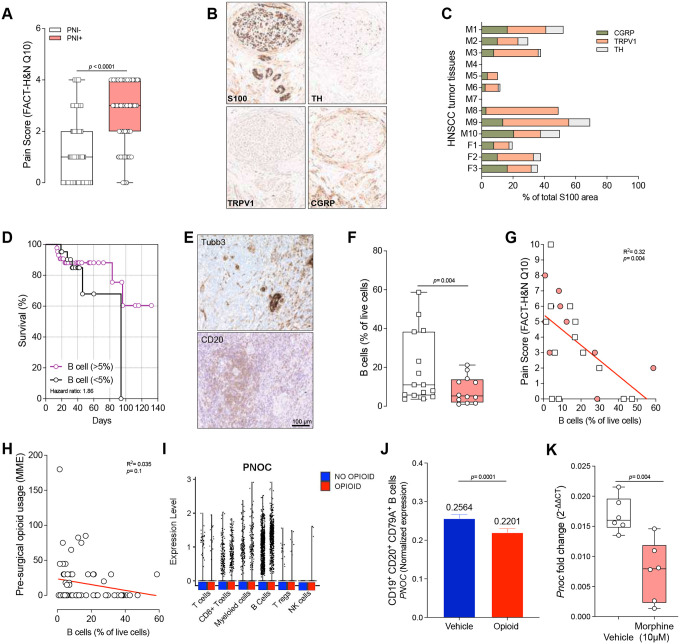
Opioids reduce *PNOC* expression and survival in head and neck squamous cell carcinoma patients. **A**, Patient-reported pain levels (measured by Functional Assessment of Cancer Therapy - Head & Neck cancer (FACT-HN) questionnaire question 10 (Q10)) were higher in head and neck squamous cell carcinoma (HNSCC) patients whose biopsy shows perineural invasion (PNI). **B-C,** Representative histological staining of a HNSCC biopsy tumor-associated neurons stain with S100^+^, TH^+^, TRPV1^+^, and CGRP^+^. **C**, HNSCC patient tumors demonstrated a higher density of nociceptor neurons (TRPV1^+^, CGRP^+^) compared to autonomic neurons (TH^+^); M = male patients (n=10); F = female patients (n=3). **D**, Cytometric evidence of B-cell density assessed in HNSCC biopsies was used to correlate the survival rate in 64 patients with HNSCC with the presence of B-cells in the tumor (assessed via flow cytometry in fresh tumor sample). Data shows the survival of patients with high (>5%) and low (<5%) B cell infiltration (Chi squared test). **E,** Cytometric evidence of B-cell density assessed in HNSCC biopsies revealed a trend in lower % B-cell in patients with PNI compared to patients without PNI. Unpaired t test with Welch’s correction due to non-normality in standard deviations. **F**, Representative histological staining illustrates neural invasion (top, pan-neuronal stain, Tubb3) and the proximity (≥ 20 μm) of CD20^+^ B cells (bottom, CD20) to tumor-infiltrating nerves. Scale=100μm. **G,** Higher reported oral pain levels (FACT-HN) in HNSCC patients negatively correlated with B-cell infiltration in their tumor biopsies (assessed via flow cytometry in fresh tumor sample), suggesting a protective role for B cells in modulating pain. **H**, HNSCC patient-reported pre-surgical opioid usage (expressed as morphine milligram equivalent (MME)) was not correlated with tumor B-cell density. **I-J**, *In silico* analysis of single-cell RNA sequencing data from HNSCC patient biopsies revealed *PNOC* expression in B cells remained unchanged following opioid treatment (**I**). But there was a lower overall proportion of PNOC-expressing B-cells (defined as CD19^+^CD20^+^CD79A^+^) in patients receiving opioids pre-surgically (**J**). **K**, Splenic B cells from naive C57BL/6 mice were cultured for 48 hours under LPS + IL-4 exposure. Cells were then treated daily for 96 hours with vehicle or morphine (10 μM). Morphine-treated cells showed reduced *Pnoc* expression. Data are shown as box-and-whisker plots (extending from the minimum to the maximum values, with the box spanning the 25th to 75th percentiles and the middle line indicating the median) (**A, E, K**), as representative images (**B, F**), as Mantel–Cox regression (**D**), as a linear regression (**G, H**), as mean ± s.e.m (**J**), or as dot plot (**I**). n as follows: **A:** 217 (PNI^neg^) and 126 (PNI^pos^); **B:** n=1 representative image; **C:** 13 patients, 10 males, 3 females; **D:** 21 (<5%) and 42 (>5%); **E:** 15 (PNI^neg^) and 12 (PNI^pos^); **F:** n=1 representative image; **G:** 23; **H:** 64; **I, J:** 2850 (vehicle) and 3044 (opioid); **K:** 6 mice. p values were determined by two-tailed Mann–Whitney test (**A, I**), Mantel–Cox regression (**D**), unpaired t-test (**E, K**), or linear regression analysis (**G, H**).

Head and neck cancer is often characterized as a neurotropic malignancy due to its high incidence of perineural invasion (**PNI**), which is associated with more severe pain and poorer survival rates^24^. In a study of 343 HNSCC patients, those with pathologically confirmed PNI reported significantly higher pain scores on the FACT-HN questionnaire^25^. Despite the extensive documentation of PNI in HNSCC, the specific nerve subtypes—motor versus sensory—that are involved remain poorly characterized. In our assessment of 13 patients (10 males, 3 females) with tongue squamous cell carcinoma, intratumoral sensory nerves expressing TRPV1 and CGRP dominated and appeared to be responsible for pain transmission (Fig. 2A–C).

Next, we examined a cohort of 64 HNSCC patients in which B cell density was quantified by flow cytometry from fresh tumor tissue. Patients with more than 5% B cell infiltration had better survival outcomes (Fig. 2D). Although B-cells were found near nerve bundles in HNSCC tumors, their infiltration was notably lower in PNI-positive tumors compared with PNI-negative tumors (Fig. 2E–F). Moreover, an inverse correlation emerged between patient-reported pain and B cell infiltration, independent of PNI status in a sub analysis of 23 patients (Fig. 2F–G), implying that B cell–mediated immune responses may influence pain perception.

Because HNSCC patients frequently experience significant pain early in the disease course, they often receive opioids prior to surgery. These exogenous opioids can activate immunosuppressive pathways and may also affect endogenous opioid expression. We found that pre-surgical opioid use, measured in morphine milligram equivalents, was not significantly correlated with B cell density in the 64 HNSCC patient cohort (Fig. 2H). However, when expanding our analysis into the in silico RNAseq dataset, we found that stratification of HSCC patients by pre-surgical opioid use revealed no change in *PNOC* expression per cell (Fig. 2I) but the overall proportion of *PNOC*-expressing B-cells diminished in patients who had received opioids (Fig. 2J). To examine this further we exposed cultured murine CD19^+^ B cells to 10 μM morphine for 4 days and found a 2.5-fold decrease in relative *Pnoc* expression compared to vehicle treated cells (Fig 2K).

### N/OFQ:OPRL1 Signaling in Pain Modulation

Although OPRL1 receptor agonists have shown analgesic effects in rodent models of neuropathic and inflammatory pain^18,26–28^, their potential impact on pain associated with HNSCC has not been fully established. We hypothesized that B cell–derived N/OFQ might bind OPRL1 on tumor-innervating sensory neurons, thereby reducing nociception (i.e. pain signaling) in a mouse model of oral squamous cell carcinoma (**oSCC**), a subtype of HNSCC.

**Fig. 3:**
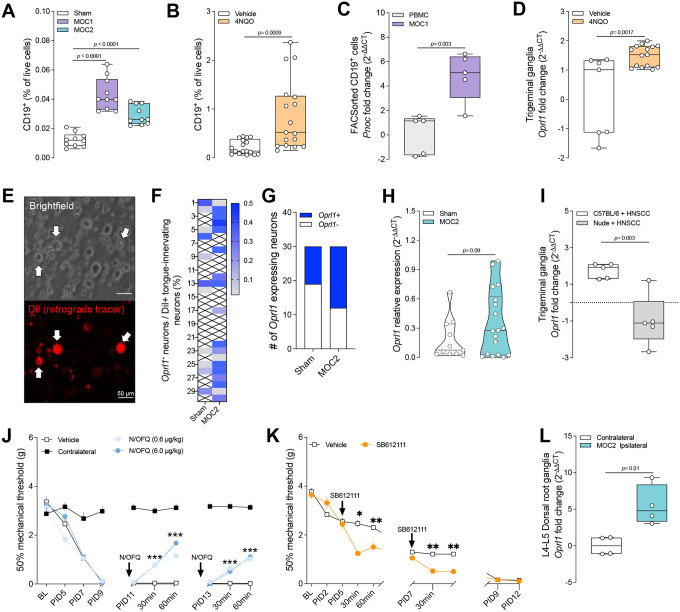
N/OFQ modulates pain responses in oral squamous-cell carcinoma (oSCC). **A,** Orthotopic oSCC cells (MOC1 (1×10^6^) or MOC2 (2×10^4^)) were injected intramuscularly into the anterior tongues of eight-week-old male and female C57BL/6 mice; sham mice received only culture media. Tongue tumors were collected at 250mm^3^ and tumor-infiltrating B cells quantified by flow cytometry. Both MOC1- and MOC2-bearing tongues showed increased infiltration of CD19^+^ B cells compared with sham controls. **B,** 4-Nitroquinolin-1-oxide (4NQO; 100 μg/ml) or vehicle (propylene glycol; 5mg/ml) was administered in the drinking water of eight-week-old male and female C57BL/6 mice for 16 weeks. Twelve weeks later, tongue tumors were harvested and CD19^+^ B cells quantified by flow cytometry; 4NQO treatment likewise increased CD19^+^ B cells infiltration relative to vehicle controls. **C,** Orthotopic oSCC cells (MOC1 (1×10^6^) or MOC2 (2×10^4^)) were injected intramuscularly into the anterior tongues of eight-week-old male and female C57BL/6 mice; sham mice received only culture media. Tongue tumors were collected at 250mm^3^ and dissociated for FACS isolation of CD19+ B cells into lysis buffer for RNA isolation. *Pnoc* levels were measured by qPCR. *Pnoc* expression was higher in CD19^+^ B cells from MOC1 tumors than in CD19^+^ B cells from sham control tongues. **D,** 4-Nitroquinolin-1-oxide (4NQO; 100 μg/ml) or vehicle (propylene glycol; 5mg/ml) was administered in the drinking water of eight-week-old male and female C57BL/6 mice for 16 weeks. Twelve weeks later, bilateral trigeminal ganglia were harvested and processed for RNA extraction. *Oprl1* expression was quantified by qPCR. Data show that *Oprl1* expression was elevated in trigeminal neurons from 4NQO-treated mice versus vehicle controls. **E–H,** Tongue-innervating neurons were labeled with the retrograde tracer DiI (170mg/ml DMSO, diluted 1:10 in saline) injection into the anterior tongue of eight-week-old male and female C57BL/6 mice. One week later, MOC2 cells (2×10^4^) were injected intramuscularly into the anterior tongues. When tongue tumors reached 250mm^3^, bilateral trigeminal ganglia were harvested, dissociated and tracer positive neurons were picked up using eletrodes for single cell PCR. To assess *Oprl1* expression. **G-H,** The proportion of *Oprl1* expressing neurons was greater in MOC2 tongue tumor mice compared to sham and there was a trend in greater relative expression. **I,** MOC1 cells (1×10^6^) were injected intramuscularly into the anterior tongue of immunocompetent (C57BL/6) or immunodeficient (NOD-*scid* IL2Rg^null^) mice, respectively. When tumors reached 250mm^3^, tongue-innervating trigeminal ganglia were collected and *Oprl1* expression was quantified by qPCR. Data show that *Oprl1* expression was higher in neurons from immunocompetent than from immunodeficient mice. **J–L,** MOC2 cells (2×10^4^) were injected into the left hind paw of eight-week-old male and female C57BL/6 mice. Mechanical hypersensitivity was assessed as the 50 % paw-withdrawal threshold of the tumor-bearing (ipsilateral) paw relative to the contralateral paw after acute intratumoral administration of (**J**) N/OFQ (0.6 μg kg^−1^ or 6 μg kg^−1^); (**K**), OPRL1 antagonist SB612111 (3mM; 33mM DMSO diluted in pH 7 PBS) **J**), or their respective vehicles. **J**, N/OFQ increased withdrawal thresholds (i.e., reduced pain) at 30 and 60 min on post-inoculation days (PID) 11 and 13. **K,** SB612111 (3mM) decreased thresholds (i.e., increased pain) at 30 and 60 min on post-inoculation days (PID) on PID 5 and 7. **L** On day 14 the mice were euthanized, ipsilateral L3–L5 dorsal-root ganglia (DRG) were collected, and *Oprl1* expression was measured by qPCR; DRG from ipsilateral MOC2-bearing paws expressed higher *Oprl1* levels than contralateral DRG. Data are shown as box-and-whisker plots (extending from the minimum to the maximum values, with the box spanning the 25th to 75th percentiles and the middle line indicating the median) (**A, B, C, D, H, K**), as representative images (**E**), as heatmap (**F**), as violin plot (**G**), or as time-course line plot (**I, J**). n as follows: **A:** 10 per group; **B:** 17 per group; **C:** 5 per group; **D:** 7 (vehicle) and 15 (4NQO); **F, H:** 11 (sham) and 18 (MOC2); **I:** 5 per group; **J:** 8 per group; **K:** 5 per group; **L:** 4 per group. p values were determined by two-sided unpaired Student’s t-test (**A, B, C, D, H, K**), unpaired Welch’s t-test (**G**), or two-way ANOVA with Bonferroni correction (**I, J**).

In an orthotopic oSCC inoculation model (using MOC1 (1×10^6^) and MOC2 (2×10^4^) cell lines), as well as in a carcinogen-induced model (4-Nitroquinolin-1-oxide, **4NQO**), we noted increased infiltration of CD19^+^ B-cells compared with controls (Fig. 3A–B). Additionally, CD19^+^ B-cells isolated from MOC1 tumors exhibited higher *Pnoc* expression than peripheral blood mononuclear cells (Fig. 3C), while trigeminal ganglia (TG) neurons from 4NQO-induced oSCC mice showed a 1.5-fold increase in *Oprl1* expression relative to controls (Fig. 3D).

Given the highly heterogeneous trigeminal innervation in the head and neck region, we performed retrograde labeling from the tongue before tumor cell inoculation and subsequently conducted single-cell PCR on labeled trigeminal neurons to assess *Oprl1* expression in tongue-innervating sensory neurons. In MOC2-bearing mice, these tracer positive-labeled neurons had a higher percentage of OPRL1^+^ cells (18/30 vs. 11/30 in sham controls) and increased relative expression of *Oprl1* (Fig. 3E–H). This upregulation depended on a functional immune system, as athymic nude mice with tumors did not exhibit comparable changes in the TG (Fig. 3I).

To assess the analgesic potential of N/OFQ in cancer-induced pain, we inoculated the hind paws of wild-type mice with MOC2 cells (2×10^4^) and measured mechanical hypersensitivity using von Frey filaments. Nociception (i.e. pain behavior) was first detected at post-inoculation day (PID) 11. A single injection of N/OFQ (0.6 or 6 μg/kg, 20 μL) transiently alleviated mechanical hypersensitivity, and a second injection on PID 13 produced a similar effect (Fig. 3J). Conversely, blocking N/OFQ signaling with the OPRL1 antagonist SB612111 (3 mM, 20 μL) further lowered mechanical thresholds on PID 5 and 7, indicating an increase in pain (Fig. 3K). Lastly, ipsilateral L3-L5 dorsal root ganglia (DRG), which contain neuronal cell bodies that innervate the hindpaw, from MOC2 tumor mice had 5-fold higher *Oprl1* expression compared to the contralateral side (Fig. 3L). These findings suggest that endogenous N/OFQ:OPRL1 signaling is initiated early during tumor development and postpones the onset of tumor-induced mechanical hypersensitivity. Neither N/OFQ nor SB612111 affected tumor cell viability (SF. 4).

### N/OFQ:OPRL1 Signaling Enhances Anti-Tumor Immunity

Using orthotopic melanoma models in rodents, we found that thermal hypersensitivity arose prior to significant tumor expansion and T-cell exhaustion^3^. In addition, and as we previously found, nociceptor neuron ablation decreased melanoma growth (SF. 5). Given the strong survival advantage tied to *OPRL1/PNOC* expression in SKCM (Fig. 1A–B, D–F) and the observation that *Pnoc* expression was restricted to B-cells (Fig. 1C, SF. 1), we next explored how *Oprl1* expression is regulated in melanoma-exposed nociceptor neurons.

**Fig. 4.**
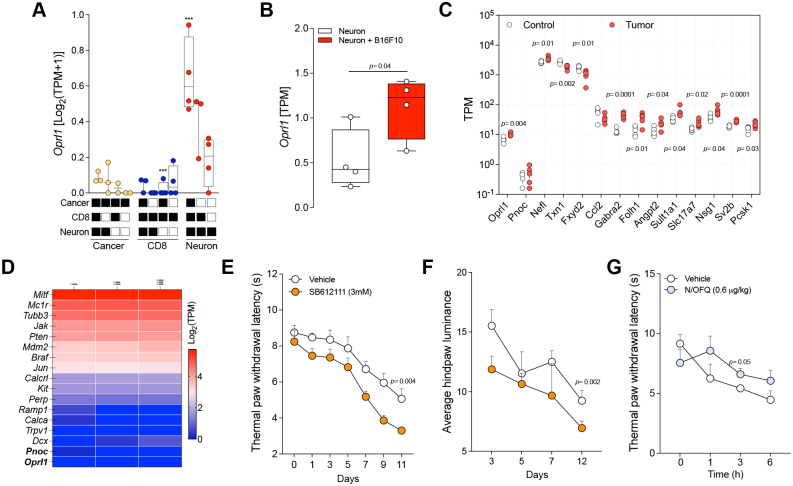
Tumor-innervating neurons express *Oprl1*, regulating pain sensitivity. **A,** *In silico* analysis of RNA-sequencing data from Balood et al., (GSE205864)^3^ in which naïve dorsal root ganglion (DRG) neurons (Trpv1^cre^::-CheRiff-eGFP^fl/wt^), B16F10-mCherry-OVA melanoma cells, and OVA-specific CD8+ T cells were cultured alone or in combination for 48 hours. After FACS purification, RNA sequencing revealed that cancer-exposed TRPV1^+^ neurons upregulate distinct gene clusters, including *Oprl1*. **B,** *In silico* analysis of RNA-sequencing data from Balood et al., (GSE205865)^3^ in which naïve DRG neurons (Trpv1^cre^::-CheRiff-eGFP^fl/wt^) were cultured alone or with B16F10-mCherry-OVA cells. After 48 hours, the cells were collected, FACS purified, and RNA-sequenced. Hierarchical clustering of differentially expressed genes (DEGs) from the sorted neurons shows distinct groups of transcripts enriched in cancer-exposed TRPV1^+^ neurons—most notably *Oprl1*. **C,** Orthotopic B16F10-OVA cells or non-tumorigenic keratinocytes (2×10^5^ cells) were injected intradermally into the left hind paw of nociceptor-reporter mice (Trpv1^cre^::tdTomato^fl/wt^). Two weeks post-injection, L3–L5 DRG neurons were harvested, TRPV1^+^ neurons were FACS-purified and RNA-sequenced. DEGs analysis revealed that *Oprl1* is overexpressed in DRG neurons from tumor-inoculated mice. **D,** *In silico* analysis of three different B16F10 cell cultures (labeled i, ii, iii) originally described by Castle et al.^57^ and re-analyzed by Balood et al.^3^ shows basal expression of *Braf* and *Pten*, but no detectable transcripts of *Pnoc* or *Oprl1*. **E, F,** Orthotopic B16F10-OVA cells (2×10^5^) were injected intradermally into the right hind paw of wild-type mice. Starting one day later, mice received daily intradermal paw injections of either vehicle (50 μL) or the OPRL1 antagonist SB612111 (3 mM, 50 μL). The evoked thermal pain hypersensitivity and the spontaneous mechanical sensitivity were assessed every other day with the Hargreaves test and the BlackBoxBio system, respectively. By day 11, OPRL1 blockade had heightened thermal hypersensitivity (**E**) and reduced paw luminance (**F**). **G,** Intradermal hind paw injection of recombinant N/OFQ (0.6 μg/kg, 20 μL) reduced the thermal pain hypersensitivity 3 hours post-injection in comparison to the vehicle injection. Data are shown as box-and-whisker plots (ranging from the minimum to the maximum values, with the box extending from the 25^th^to the 75^th^percentile and the middle line indicating the median), for which individual data points (**A, B**), as scatter dot plots with medians in (**C**), as heatmap displaying gene expression as log_2_ transcripts per million (TPM) (**D**), and as mean ± s.e.m. (**E, F, G**). n as follows: **A:** 3–4 per group; **B:** 4 per group; **C:** 5 per group, **D:** 3; **E, F:** 11 (vehicle) and 12 (SB12111); **G:** 6 per group. p values were determined by nested one-way ANOVA with post hoc Tukey (**A**), two-sided unpaired Student’s t-test (**B, C**), or two-way ANOVA with post hoc Bonferroni (**E, F, G**).

By analyzing our previously published datasets *in silico*, we discovered that naïve DRG neurons co-cultured with B16F10-mCherry-OVA melanoma cells and OVA-specific CD8^+^ T-cells exhibited elevated *Oprl1* expression in cancer-exposed TRPV1^+^ neurons (Fig. 4A). We confirmed this in a separate bulk RNA-seq dataset in which neurons were co-cultured with or without B16F10 cells (Fig. 4B). Building on these observations, we examined whether this cancer-driven neuronal plasticity also occurs *in vivo*, as observed in our head and neck cancer models (Fig. 3G, H, L). For this *in vivo* test, we inoculated either B16F10-OVA melanoma cells or non-tumorigenic keratinocytes (2×10^5^ cells) into the left hind paws of Trpv1^cre^::td-tomato^fl/wt^ nociceptor-reporter mice. Two weeks later, we harvested L3–L5 DRG neurons, TRPV1^+^ cells (tdTomato^+^) were FACS-purified and analyzed by RNA-seq. Differential gene expression analysis showed that *Oprl1* was overexpressed in DRG neurons from tumor-bearing mice (Fig. 4C).

Next, we investigated whether N/OFQ:Oprl1 signaling controls melanoma-induced pain. Orthotopic B16F10-OVA melanoma cells (2×10^5^) were inoculated into the right hind paws of wild-type mice, which subsequently received daily intradermal injections of vehicle (50 μL) or the OPRL1 antagonist SB612111 (3 mM; 50 μL), starting one day post-inoculation. Consistent with our prior findings, melanoma evoked a gradual increase in hindpaw thermal hypersensitivity, measured by the Hargreaves method and BlackBoxBio, a user-independent spontaneous pain sensor (Fig. 4E–F). Compared with vehicle-treated mice, SB612111 further intensified thermal hypersensitivity, peaking on day 11 post-inoculation (Fig. 4E–F) suggesting an active N/OFQ:OPRL1 signal ongoing during melanoma progression. We then tested whether additional activation of OPRL1 with recombinant N/OFQ (0.6 μg/kg; 50 μL) could alleviate melanoma-induced pain. As anticipated, on the peak pain day (**PID** 11), thermal hypersensitivity was significantly reduced within three hours of N/OFQ administration (Fig. 4G).

### Disrupting OPRL1 Signaling Exacerbates Pain and Tumor Progression

Collectively, our findings indicate that melanoma and head and neck tumors are innervated by neurons upregulating *Oprl1* (Fig. 3H, Fig. 4C), and these tumors also harbor *Pnoc*-expressing B-cells (Fig. 3A–C). We showed that activating the N/OFQ:OPRL1 axis reduces cancer pain, whereas blocking it worsens pain. In line with our previous research demonstrating that pain precedes and drives immunosuppression in multiple cancers (including melanoma and head and neck malignancies)^3,4^ and that pain neuron control tumor growth (SF. 5), we hypothesized that *PNOC*-expressing B-cells in the tumor microenvironment could inhibit CGRP-expressing nociceptors, thereby promoting anti-tumor immunity.

To explore this hypothesis, we first examined patient immune infiltration signatures in various cancers. We observed that *PNOC* expression strongly correlated with more robust anti-tumor immune responses, characterized by enhanced CD8^+^ T-cells and B-cells infiltration, as well as decreased levels of immunosuppressive subsets such as myeloid-derived suppressor cells (**MDSCs**) (SF. 7, 8). Notably, we found a strong correlation (FDR = 4.5e^−54^) between *PNOC* expression and the B-cell infiltration signature (SF. 8A). Moreover, among head and neck cancer patients, the HPV^+^ subgroup—known for lower pain^25^—displayed higher levels of *PNOC*-expressing B-cells than HPV^−^ patients (SF. 9).

**Fig. 5.**
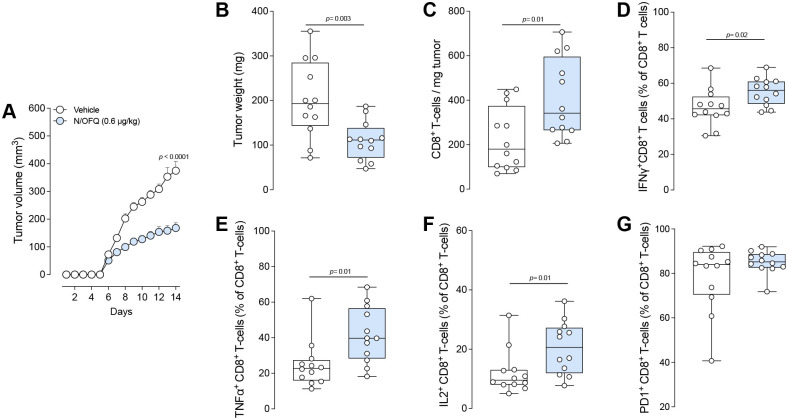
OPRL1 activation reduces tumor growth and improves anti-tumor immunity. **A-G**, Orthotopic B16F10-OVA cells (5×10^5^) were inoculated intradermally into the flank of wild-type mice. Starting one later, mice received daily intradermal injections of either vehicle (50 μL) recombinant N/OFQ (0.6 μg/kg; 50 μL) at five sites around the tumor. **A, B,** Fourteen days after tumor inoculation, recombinant N/OFQ administration significantly reduced tumor growth (**A**), and tumor weight (**B**). **C-G**, Immunophenotyping of tumor-infiltrating cells revealed increased total CD8^+^ T-cells (**C**) and proportion of CD8^+^ T-cells expressing IFNγ^+^ (**D**), TNFα^+^ (**E**), and IL2^+^ (**F**) in mice treated with N/OFQ. No change was observed in the proportion of PD1^+^ CD8^+^ T-cells (**G**). Data are shown as mean ± s.e.m. in (**A**) or as box-and-whisker plots with individual data points in (**B-G**). n as follows: 12 per group. p values were determined by two-way ANOVA with post hoc Bonferroni (**A**) or two-sided unpaired Student’s t-test (**B-G**).

**Fig. 6.**
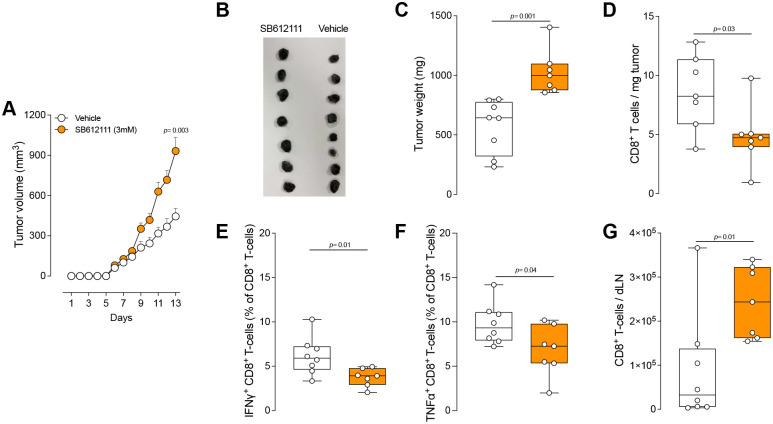
OPRL1 blockade impairs immunosurveillance and increases tumor growth. **A-G,** Orthotopic B16F10-OVA cells (5 × 10^5^ cells, i.d.) were inoculated into the flank of wild-type mice. Starting one day later, mice received daily intradermal injections of either vehicle (50 μL) or the OPRL1 antagonist SB612111 (3 mM; 50 μL) at five sites around the tumor. Thirteen days after tumor inoculation, mice treated with SB612111 showed increased tumor volume (**A, B**), and tumor weight (**C**). Immunophenotyping of tumor-infiltrating cells revealed decreased infiltration of CD8^+^ T-cells (**D**), and proportion of CD8^+^ T-cells expressing IFNγ^+^ (**E**), and TNFα^+^ (**F**) in mice treated with OPRL1 antagonist. OPRL1 antagonist treatment increased the number of CD8^+^ T-cells in tumor-draining lymph nodes (dLN) (**G**). Data are shown as mean ± s.e.m. (**A**), as representative image (**B**), and as box-and-whisker plots with individual data points indicated (**C-G**). n as follows: **A-G**: 8 (vehicle) and 7 (SB612111). p values were determined by two-way ANOVA with post hoc Bonferroni (**A**) or two-sided unpaired Student’s t-test (**C-G**).

Next, we tested this relationship using B16F10 orthotopic melanoma model and found that the local administration of N/OFQ (0.6 μg/kg) during tumorigenesis significantly slowed tumor growth and reduced tumor weight by PID 14 (Fig. 5A–B). Flow cytometric analysis revealed more cytotoxic CD8^+^ T-cells (Fig. 5C) with higher IFNγ (Fig. 5D), TNFα (Fig. 5E), and IL2 (Fig. 5F) expression—indicative of a more effective anti-tumor immune response (SF. 8). Consistent with the beneficial effects of endogenous N/OFQ:OPRL1 signaling in oSCC models, we hypothesized that blocking OPRL1 would undo N/OFQ’s benefits. Indeed, local administration of the OPRL1 antagonist SB612111 (3 mM) to tumor-bearing mice (both flank and hind paw models) accelerated tumor growth and increased tumor weight by PID 13 (Fig. 6A–C, SF. 11). Immunophenotyping of flank tumors demonstrated fewer CD8^+^ cytotoxic T-cells (Fig. 6D) with reduced IFNγ (Fig. 6E) and TNFα (Fig. 6F) expression. Additionally, there was an accumulation of CD8^+^ T-cells with diminished cytokine levels in tumor-draining lymph nodes, suggesting hindered T cell migration or recruitment (Fig. 6G).

Neither exogenous N/OFQ nor SB612111 altered B16F10 cell viability (SF. 6). Furthermore, cancer cells did not modify their *Pnoc* or *Oprl1* expression in the absence of nociceptor neurons, indicating that N/OFQ signaling primarily regulates neuronal activity and, in turn, affects tumor–neuron communication and immune responses. Overall, these findings confirm that N/OFQ signaling through OPRL1 suppresses nociceptor activity while reshaping the tumor immune environment, offering a novel approach to managing both cancer pain and anti-tumor immunity.

### Reciprocal Neuroimmune Crosstalk in Melanoma Progression

**Fig. 7.**
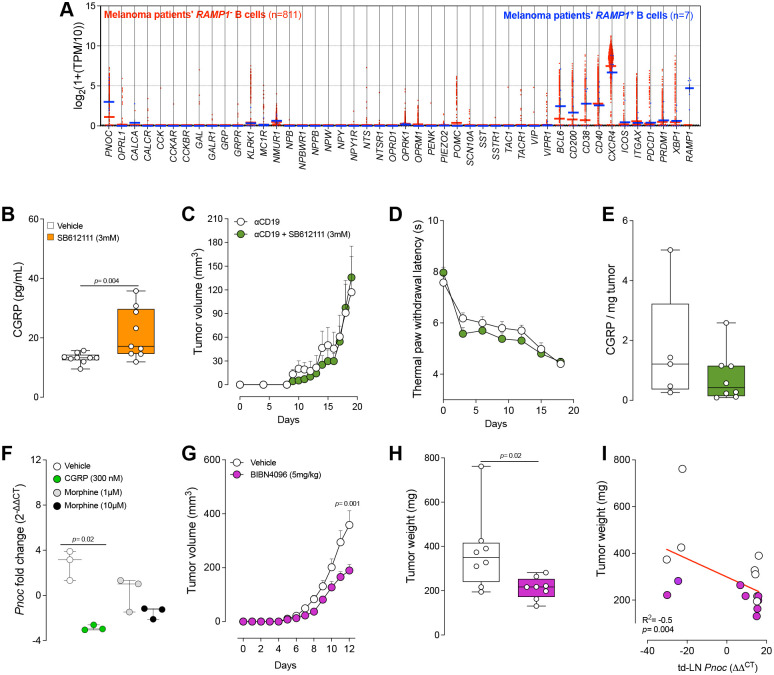
CGRP regulates *PNOC* expression and tumor growth. **A,** *In silico* analysis of single-cell RNA sequencing data (GSE115978)^55^ from skin cutaneous melanoma (SKCM) patient biopsies displays that intratumoral *RAMP1*-expressing B-cells showed substantially higher *PNOC* expression compared to *RAMP1*-negative B-cells. **B,** Orthotopic B16F10-OVA cells (2×10^5^) were inoculated into the right hind paw of wild-type mice. Starting one later, mice received daily intradermal injections of vehicle (50 μL) or the OPRL1 antagonist SB612111 (3 mM; 50 μL). On day 11, tumor explants were harvested, cultured, and exposed to capsaicin to induce peptide release. Conditioned media were then collected and assessed for CGRP by ELISA. SB612111-treated mice tumors released higher levels of CGRP. **C-E,** To deplete circulating B-cells, 8-week-old C57BL/6 male and female mice were treated with anti-CD19 (αCD19; 200 μg) once a week for three consecutive weeks. Subsequently (5 days later), the mice were inoculated in the right hind paw with B16F10-OVA cells (2×10^5^). Starting one day later, mice received daily intradermal injections of vehicle (50 μL) or SB612111 (3 mM; 50 μL). Under B-cell–depleted conditions, OPRL1 blockade did not affect tumor growth (**C**). The evoked thermal pain hypersensitivity was assessed every three days with the Hargreaves test. OPRL1 blockade did not alter tumor-induced thermal hypersensitivity (**D**). On day 20, tumor explants were harvested, cultured, and exposed to capsaicin to induce peptide release. Conditioned media were then collected and assessed for CGRP by ELISA. OPRL1 blockade had no effect on CGRP release in B-cell–depleted mice (**E**). **F,** Splenic B cells from naive C57BL/6 mice were cultured for 48 hours under LPS + IL-4 exposure. Cells were then treated daily for 96 hours with vehicle or CGRP (300 nM). CGRP-treated cells showed reduced *Pnoc* expression. **G-I,** Orthotopic B16F10-OVA cells (2×10^5^) were inoculated intradermally into the flank of wild-type mice. Starting one day later, mice received intraperitoneal injections of vehicle (100 μL) or the RAMP1 antagonist BIBN4096 (5 mg/kg) every other day. By day 12, BIBN4096 significantly reduced tumor growth (**G**) and tumor weight (**H**). Higher tumor weight negatively correlated with *Pnoc* expression in the tumor-draining lymph nodes (td-LN) (**I**). Data are shown as violin plots (**A**), as box-and-whisker plots (extending from the minimum to the maximum values, with the box spanning the 25^th^to 75^th^percentiles and the middle line indicating the median), where individual data points are shown (**B, E, H**), as mean ± s.e.m.: (**C, D, G**), as scatter dot plots with medians (**F**) or as mantel-cox regression (**I**). n as follows: **A:** 811 (Ramp1^neg^) and 7 (Ramp1^pos^); **B:** 9 (vehicle) and 7 (SB612111); **C:** 5 (αCD19) and 9 (αCD19 + SB612111); **D, E:** 5 (αCD19) and 8 (αCD19 + SB612111); **F:** 3 per group; **G-I:** 8 per group. p values were determined by two-sided unpaired Student’s t-test (**A, B, E, H**), one-way ANOVA with Bonferroni post hoc (**F**), two-way ANOVA with Bonferroni post hoc (**C, D, G**), or simple linear regression (**l**).

Because nociceptor neurons modulate B-cell activity in diverse contexts—such as lymph nodes during infection^3^, lungs in allergic inflammation^29,30^, and within tertiary lymphoid structures in tumor-bearing mice^31^— we investigated whether CGRP-expressing neurons and *Pnoc*-expressing B-cells engage in crosstalk during melanoma progression. Supporting this view, single-cell RNA-seq data for SKCM revealed a population of B cells expressing CGRP receptor component, *RAMP1*, that markedly overexpressed *PNOC* compared with *RAMP1*-negative B-cells (Fig. 7A) suggesting that CGRP-expressing nociceptors could directly interact with B cells (Fig. 7A).

Since OPRL1 blockade increased tumor-induced pain (Fig. 4E–F) and impaired anti-tumor immunity (Fig. 6), we asked whether it also heightens intra-tumoral neuronal activity. Indeed, blocking OPRL1 raised intra-tumoral CGRP levels (Fig. 7B). To ascertain if B cell infiltration mediates this N/OFQ:OPRL1 effect, we depleted B-cells with an anti-CD19 antibody in a B16F10 melanoma model. In these B cell–depleted mice, OPRL1 antagonism (**SB612111**) no longer influenced tumor growth, nociceptive behavior, or CGRP levels (Fig. 7C–E), indicating that B-cells are crucial for the antinociceptive and anti-tumor effects of N/OFQ. Furthermore, when splenic CD19^+^ B-cells were cultured in the presence of CGRP (300 nM), LPS, and IL4, *Pnoc* expression declined three-fold relative to controls (Fig. 7F). These findings suggest that nociceptor-derived CGRP reciprocally modulate B cell function.

Because CGRP strongly suppresses cytotoxic CD8^+^ T-cells and can also shape B cell activity, we tested the CGRP receptor antagonist BIBN4096, which significantly slowed tumor growth (Fig. 7G–H) and altered the phenotype of *Pnoc*-expressing B-cells. Finally, we detected an inverse relationship between tumor weight and *Pnoc* expression in B-cells from tumor-draining lymph nodes (Fig. 7I). Together, these results emphasize a reciprocal neuroimmune interaction in the tumor microenvironment, whereby CGRP signaling from sensory neurons modulates B cell function, ultimately regulating both nociception and tumor control (SF 12). Targeting the N/OFQ:OPRL1 pathway represents a promising therapeutic strategy for simultaneously alleviating cancer pain and impeding tumor progression.

## DISCUSSION.

Melanoma and head and neck squamous cell carcinoma (**HNSCC**) both arise from the ectoderm: melanoma from neural crest–derived melanocytes and HNSCC from ectoderm-derived epithelial cells. Peripheral sensory neurons share this lineage, suggesting that they may express similar receptors and neurotransmitters. Such convergence could shape tumor behavior and responses to therapy. Melanoma is highly aggressive with substantial metastatic potential. Despite earlier detection and targeted therapies, outcomes in advanced stages remain poor. HNSCC, which makes up more than 90% of head and neck cancers, also tends to be diagnosed at later stages, with a 50% recurrence rate within the first 2 years. Additionally, HPV-associated HNSCC has changed prognostic patterns, emphasizing the need for innovative therapeutic strategies^32,33^.

The immune system recognizes and destroys aberrant cells, including cancer. However, tumors often exploit sustained receptor signaling in immune cells, leading to T-cell exhaustion and immune escape. Traditional therapies, even targeted ones, may offer limited benefits. While immunotherapies have revolutionized treatment, not all patients respond. Tumor-infiltrating neurons have emerged as critical factors that modulate local immunity. Cancer cells secrete growth factors that induce hyperinnervation^34–36^, and many tumors present with significant pain^7,37^. Disrupting nerve supply—for example, via surgery in head and neck cancer^38^, Botox in prostate cancer^39,40^, or vagotomy in gastric ulcer patients^41,42^—slows tumor growth and reduces cancer mortality rates. Although the mechanisms remain incompletely defined, pain-sensing nociceptor neurons can promote tumor progression by altering cytokine release, stimulating angiogenesis, or aiding metastasis^3,43–47^.

In melanoma, peripheral sensory neurons in the tumor microenvironment enhance tumor growth and block tertiary lymph node formation^31^. Nociceptors release calcitonin gene–related peptide (**CGRP**), which drives cytotoxic T-cell exhaustion and decreases tumor clearance. Blocking the CGRP receptor RAMP1 revives T-cell activity^3,4^. Germline CGRP-knockout mice with head and neck tumors show reduced tumor size, greater infiltration by cytotoxic CD8^+^ T-cells and NK cells, and improved responsiveness to radiation^1^. CGRP is also induced in low-glucose areas typical of oral melanoma and mucosal carcinomas, where it promotes protective autophagy in cancer cells^2^ and fosters resistance to anti-PD-1 therapy^3^. Although opioids remain the standard therapy for cancer pain^37^, they can have ambiguous effects on tumor biology: at lower doses they may facilitate tumor progression and immunosuppression, while higher doses appear to have antiangiogenic and pro-apoptotic actions^7^.

Our work highlights a central role for B cell–derived nociceptin/orphanin FQ (**N/OFQ**) in both melanoma and HNSCC. Patient datasets and tumor-bearing mouse models show that N/OFQ not only dampens pain but also fortifies immunosurveillance and hinders tumor progression, underscoring its potential as a therapeutic agent.

### N/OFQ and pain modulation

N/OFQ is widely recognized for its role in pain regulation. Evidence in rodent models demonstrates that N/OFQ exerts a bimodal modulation of pain acting through central and peripheral mechanisms; analgesic effects are produced through the spinal pain pathways whereas anti-opioid hyperalgesic effects are possible when acting within the brain^12,48^. Our paw withdrawal and Hargreaves tests reveal that N/OFQ can modulate nociceptive signaling but is dependent on both dosage and tumor environment. OPRL1 agonists have emerged as a promising target for developing novel and effective opioids that modulate the analgesic and addictive properties of OPRM1 agonists. Selective OPRL1 agonists have been tested in non-human primates and found to produce full analgesia when delivered systemically. Furthermore, these agonists do not induce positive reinforcement in the central nervous system, indicating a lack of abuse liability^28,49^. By carefully calibrating N/OFQ administration, balanced coactivation of OPRL1 and mu opioid receptors is a strategy that warrants further exploration and refinement to manage cancer pain, thereby limiting opioid-associated side effects.

### N/OFQ’s anti-tumor effects

In our melanoma models, N/OFQ therapy significantly reduced tumor size and increased T-cell infiltration, supporting a mechanism involving enhanced immunosurveillance rather than direct effects on cancer cell apoptosis. Earlier work in small-cell lung cancer suggested that N/OFQ can promote inflammatory pathways that favor tumor growth^20^, highlighting its context dependence. Critically, tumor-bearing mice depleted of neurons failed to show changes in N/OFQ or *Oprl1* gene expression, indicating that N/OFQ-driven antitumor activity is closely tied to nerve-cancer interactions.

### Interaction with CGRP and T-cells

Tumor-associated nociceptors secrete CGRP, which accelerates T-cell exhaustion and promotes cancer progression^3^. Our experiments show that N/OFQ can inhibit CGRP release, through inhibitory GPCR signaling at the tumor innervating terminals; limiting CGRP neurotransmission prevents the previously described RAMP1-mediated immunosuppression. Although some studies propose that N/OFQ suppresses CD4^+^ T-cell proliferation^50^, we observed increased numbers of tumor-infiltrating CD8^+^ T cells, a finding usually correlated with better clinical outcomes. Our data suggests that B cells synthesize and secrete N/OFQ, contributing to T cell–mediated tumor control. Human studies suggest that B cells within or around tumors correlate with better prognosis and enhanced responses to immunotherapy^23^. While activated B cells are indicative of a robust T cell response^23^, their direct role in therapeutic outcomes is still to be fully understood. Further research is necessary to explore how B cells interact with other cells in the tumor environment to maximize their therapeutic impact.

### Clinical relevance of the N/OFQ:OPRL1 pathway

Unbiased survival analysis indicates that high N/OFQ:OPRL1 activity correlates with better survival in HNSCC and skin cutaneous melanoma (SKCM) but may worsen outcomes in hepatocellular carcinoma. These discrepancies underscore the context-specific nature of N/OFQ signaling, influenced by cancer subtype, local concentration, and other microenvironmental variables. Targeting the N/OFQ:OPRL1 pathway could simultaneously alleviate cancer-related pain, inhibit tumor growth, and bolster immunosurveillance. N/OFQ might serve as an opioid alternative, and because it curbs T-cell exhaustion—a major barrier in cancer immunotherapy—it may be particularly advantageous when combined with checkpoint inhibitors.

### Future directions

Further research is needed to clarify the dose-response effects of N/OFQ in different malignancies and to identify molecular switches that toggle its pro- or anti-inflammatory states. Understanding how N/OFQ shapes nerve-cancer crosstalk is crucial for optimizing its therapeutic potential. Clinical trials testing both monotherapy and combination therapies involving the N/OFQ:OPRL1 axis are warranted to gauge efficacy, safety, and side-effect profiles. Overall, harnessing the pleiotropic functions of N/OFQ may yield therapies that not only relieve pain but also limit tumor progression and reinforce immunosurveillance—key goals in integrated cancer care.

## MATERIALS AND METHODS

### Single-Cell RNA Sequencing (scRNA-Seq) Analysis

scRNA-Seq data from tumor-infiltrating lymphocytes (TILs) of head and neck squamous cell carcinoma (HNSCC) patients (GSE139324) included 16 opioid-naive and 10 opioid-exposed patient samples. All data were processed using the 10X Genomics Cell Ranger pipeline. Raw gene expression matrices for each patient were converted into Seurat objects, normalized, and integrated with the Seurat package in R. Principal component analysis (PCA) was performed, and clustering was conducted on the first 20 principal components at a resolution of 0.5. Immune cell clusters were visualized with UMAP and annotated using gene markers from PanglaoDB. Feature and violin plots generated with the Plotly package were used to examine OPRM1 expression across these immune cell clusters.

### HNSCC Retrospective Patient Analysis

Patient-reported pain and peripheral nerve presence were assessed in HNSCC patients who underwent surgical resection at the University of Pittsburgh Medical Center between 2015 and 2018. All patients were over 18 years old, had squamous cell carcinoma of the oral cavity or oropharynx, and were confirmed to have perineural invasion (PNI) by an oral and maxillofacial pathologist. Patient-reported pain was measured using item 12 of the Functional Assessment of Cancer Therapy – Head and Neck (FACT-HN version 4), which states: “I have pain in my mouth, throat, or neck.” Tumor tissues were obtained from the UPMC Hillman Cancer Center’s Head and Neck SPORE (P50CA097190) tissue collection. All participants provided written informed consent, and the study was approved by the Institutional Review Board of the University of Pittsburgh Cancer Institute (STUDY20050058).

### HNSCC Tumor Tissue and Mouse Tumor Tissue Immunohistochemistry

Paraffin-embedded HNSCC patient tumor blocks were acquired from the Head and Neck Tissue Bank, sectioned (5 μm), and stained by the Developmental Pathology Laboratory in the Department of Pathology. For mouse samples, adult C57Bl/6 mice bearing MOC2 tumors (post-inoculation day 14) were anesthetized with 3–5% isoflurane and perfused transcardially with phosphate-buffered saline (PBS), followed by 20 mL of cold 4% paraformaldehyde (PFA, Electron Microscopy Sciences). Tongue tissues were harvested, bisected sagittally, and fixed in 4% PFA at 4 °C for up to 1 week before being washed in PBS, paraffin-embedded, and sectioned at 5 μm.

For both human and mouse tissues, antigen retrieval was performed using citrate buffer (Dako, Carpinteria, CA). S100 antibody (rabbit polyclonal, Cat# IR50461–2, Dako/Agilent) was applied at 1:200 dilution at room temperature. Secondary antibody labeling was performed with SignalStain Boost (HRP, Rabbit, Cell Signaling, Danvers, MA), and 3,3-Diaminobenzidine+ (Dako) was used as the chromogen. Slides were counterstained with Hematoxylin (Dako). Sections were scanned at 10×, 20×, and 40× magnification. The S100-immunoreactive area was quantified using Aperio ImageScope software (Leica Biosystems). CGRP-immunoreactive area was quantified within each S100+ nerve bundle, and the percentage of CGRP+ area relative to total S100+ area was calculated.

### Animals

Adult male and female C57BL/6 mice (6–12 weeks old, 20–30 g; stock #000664, Jackson Labs, Bar Harbor, ME) were used for all experiments. Mice were housed in a temperature-controlled room with a 12:12-hour light/dark cycle (lights on from 07:00–19:00) and provided ad libitum access to food and water. All researchers were trained under the Animal Welfare Assurance Program. Procedures were approved by the University of Pittsburgh Institutional Animal Care and Use Committee (Protocol #: 23093881) and adhered to the National Institutes of Health Guide for the Care and Use of Laboratory Animals; and by Queen’s University (Protocol #: 2023–2380) and adhered to the Canadian Council of Animal Care. The ARRIVE Essential 10 guidelines were followed for all preclinical experimental designs.

### Housing of mice

Mice were housed in standard environmental conditions (12-h light–dark cycle; 23 °C; food and water ad libitum) at facilities accredited by the Canadian Council of Animal Care (Queen’s University) or the Institutional Animal Care and Use (University of Pittsburgh).

### Animals end-points

As per approved protocol, the following endpoints were used in all of the experiments and were not exceeded. Along with excessive body weight loss (maximum of 10%), the end-points include excessive tumour volume, skin ulceration, necrosis, bleeding, infection and self-inflicted injury, prostration, lethargy, unresponsiveness to stimulation and/or lack of grooming.

### Cell lines

Mouse oral squamous cell carcinoma lines MOC1 and MOC2 (Kerafast) were grown in IMDM/F12 medium (2:1 ratio; Thermo Fisher Scientific) supplemented with 5% fetal bovine serum (FBS, Corning), 1% penicillin-streptomycin (Thermo Fisher Scientific), 5 μg/mL insulin (Sigma-Aldrich), 40 ng/mL hydrocortisone (Sigma-Aldrich), and 5 ng/mL epidermal growth factor (EMD Millipore). Cells were maintained in 10 cm culture dishes at 37 °C with 5% CO2 and used at passages below 19. B16F10-mCherry-OVA cells (provided by M.F. Krummel, UCSF) were cultured in complete Dulbecco’s modified Eagle’s medium (DMEM; Corning, 10–013-CV) supplemented with 10% FBS (Seradigm, 3100) and 1% penicillin–streptomycin (Corning, MT-3001-Cl), and maintained at 37 °C in a 5% CO2 humidified incubator.

B16F10-OVA and non-tumorigenic keratinocytes (CellnTEC, MPEK-BL6100) were cultured in complete Dulbecco’s modified Eagle’s medium high glucose (DMEM; Corning, 10–013-CV) supplemented with 10% fetal bovine serum (FBS; Seradigm, 3100) and 1% penicillin–streptomycin (Corning, MT-3001-Cl), and maintained at 37 °C in a humidified incubator with 5% CO2.

All the cell lines tested negative for mycoplasma, and none are listed by the International Cell Line Authentication Committee registry (v.11). Non-commercial cell line (B16F10-OVA) was authenticated using antibody against OVA and imaging as well as morphology and growth properties.

### Mouse Tissue Immunohistochemistry

Mice under 3–5% isoflurane were perfused transcardially with PBS, followed by 4% PFA. Tongues were dissected, bisected, and postfixed in 4% PFA for 24 hours. Samples were then cryoprotected in 30% sucrose at 4 °C overnight, embedded in Tissue-Tek OCT compound (Sakura Finetek), sectioned at 20 μm, and mounted on Superfrost Plus slides (Fisher Scientific). Sections were incubated overnight at room temperature with a mouse anti-CD19 monoclonal antibody (1:100, eBiosciences) in PBS containing 1% bovine serum albumin. After washing in PBS, slides were incubated with Alexa Fluor 800 or Alexa Fluor 647 secondary antibodies (1:250, Jackson ImmunoResearch) for 2.5 hours, then cover-slipped with Fluoro-Gel II mounting media containing DAPI (Electron Microscopy Sciences). Images were acquired at 4× to 20× magnification using a Leica DMi8 microscope with LAS software.

### Mouse B Cell Culture

Naïve CD19^+^ B cells were magnetically isolated from wild-type mouse spleens using the StemCell negative isolation CD19 kit. The B cell culture medium was prepared by combining 430 mL RPMI 1640, 5 mL sodium pyruvate (Gibco 11360–70), 5 mL 1 M HEPES buffer (Fluka 51558), 5 mL penicillin/streptomycin (Cellgro MT-3001-CI), 30 mL heat-inactivated FBS, and 1.8 μL β-mercaptoethanol. The isolated CD19+ B cells were resuspended in this medium at 25°C and adjusted to a concentration of 1×10^7^ cells/mL. A minimum of 1×10^6^ cells were added to each well of a 12-well tissue culture plate containing 2 mL of RPMI-COM medium supplemented with a stimulation cocktail (10 μg/mL LPS and 20 ng/mL IL4), alongside either vehicle or one of the following drug treatments: 1–10 μM morphine (Covetrus, stock: 1.38 mM in saline) or 300 nM αCGRP (Fisher Scientific, stock: 100 μM in dH_2O). Cells were incubated at 37°C with 5% CO_2_ for 4 days. At the end of the incubation, cells were collected, pelleted (500 × g, 4 min), snap-frozen, and processed for quantitative real-time PCR.

### Oral Cancer Mouse Model

To establish the syngeneic orthotopic oral cancer model or the hind paw oral cancer model, adult male and female mice under 3–5% isoflurane anesthesia were injected with 7.5×10^5^ MOC1 cells or 2×10^4^ MOC2 cells in 30 μL of a 1:1 mixture of DMEM (Gibco) and Matrigel (Corning) into either the anterior lateral tongue or the glabrous skin of the hind paw. Injections of DMEM/Matrigel alone served as a sham control. After injections, mice were returned to their home cages, with females housed 3–5 per cage and males housed 3–4 per cage; tumor-bearing mice were not housed with sham animals.

For the carcinogen-induced oral cancer model, mice received drinking water containing 4-nitroquinoline-1-oxide (4NQO; 100 μg/mL; Sigma Aldrich, St. Louis, MO, USA) or vehicle (propylene glycol) for 16 weeks. Mice were then monitored weekly under light anesthesia for an additional 12 weeks to assess tumor incidence, location, and size. Pilot studies showed no sex differences in the N/OFQ-induced antinociception response, so groups were evenly allocated by sex. Mice were excluded from analysis if they did not survive until the planned euthanasia date or if an open tongue lesion/injury (likely from biting) was detected in the tumor. Harvest dates varied according to experimental design. For tissue collection, mice under 3–5% isoflurane anesthesia were transcardially perfused with PBS and processed for subsequent experiments.

For single-cell PCR analysis of tongue-innervating neurons, retrograde labeling was performed at least 10 days before any experimental manipulation (e.g., tumor cell inoculation, sham injection). The retrograde tracer 1,1′-dioctadecyl-3,3,3′,3′-tetramethylindocarbocyanine perchlorate (DiI; Invitrogen, Carlsbad, CA) was prepared at 170 mg/mL in DMSO, diluted 1:10 in sterile saline, and injected bilaterally into the anterior lateral tongue (5–7 μL total per tongue) using a 30-gauge needle and Hamilton syringe under isoflurane anesthesia. This tracer labels tongue-innervating trigeminal ganglia sensory neurons.

### Cancer Inoculation and Volume Measurement

Cancer cells were resuspended in phosphate-buffered saline (PBS; Corning, 21040CV) and injected intradermally into the right flank (5×10^5^ cells in 100 μL) or the hind paw (2×10^5^ cells in 50 μL). Tumor growth was assessed daily using a handheld digital caliper, and tumor volume was calculated as (L × W2 × 0.52), where L is the length and W is the width of the tumor^64^.

### Tumor and Tumor-Draining Lymph Node Digestion

Mice were euthanized when the tumor reached a volume of 800–1,500 mm^3^. Tumors and their draining lymph nodes were collected. Tumors were enzymatically digested in DMEM + 5% FBS (Seradigm, 3100) supplemented with 2 mg/mL collagenase D (Sigma, 11088866001), 1 mg/mL collagenase IV (Sigma, C5138–1G), and 40 μg/mL DNAse I (Sigma, 10104159001) under constant shaking (40 min, 37 °C). The cell suspension was centrifuged at 400 × g for 5 min, the pellet was resuspended in a 70% Percoll (GE Healthcare) solution and overlaid with 40% Percoll. The gradient was centrifuged at 500 × g for 20 min at room temperature with the acceleration and deceleration set to 1. Cells were collected from the Percoll interface, passed through a 70-μm cell strainer, and washed. Tumor-draining lymph nodes were harvested in PBS + 5% FBS, mechanically dissociated using a plunger, strained through a 70-μm filter, and washed with PBS before further analysis.

### Immunophenotyping

Single cells were resuspended in FACS buffer (PBS, 2% fetal calf serum and EDTA), and stained with ZombieAqua (15 min, room temperature; BioLegend, 423102). The cells were washed and Fc-blocked (0.5 mg ml−1, 15 min, 4 °C; BD Biosciences, 553141). Finally, the cells were stained (30 min, 4 °C) with one of anti-CD45–BV421 (1:100, BioLegend, 103134), anti-CD45-Alexa Fluor 700 (1:100, BioLegend, 103128), anti-CD11b-APC/Cy7 (1:100, BioLegend, 101226), anti-CD8-AF700 (1:100, BioLegend, 100730), anti-CD8–BV421 (1:100, BioLegend, 100753), anti-CD8–PerCP/Cyanine5.5 (1:100, BioLegend, 100734), anti-CD8–Pacific Blue (1:100, BioLegend, 100725), anti-PD-1–PE-Cy7 (1:100, BioLegend, 109110), washed and analysed using a BD FACSymphony or a Beckman CytoFLEX.

### Intracellular Cytokine Staining

Cells were stimulated for 3 hours with phorbol-12-myristate 13-acetate (PMA, 50 ng/mL; Sigma-Aldrich, P1585), ionomycin (1 μg/mL; Sigma-Aldrich, I3909), and Golgi Stop (1:100; BD Biosciences, 554724). After fixation and permeabilization (1:100, BD Biosciences, 554714), cells were stained with the following antibodies:

- anti-IFNγ–APC (1:100; BioLegend, 505810)
- anti-IFNγ–FITC (1:100; BioLegend, 505806)
- anti-TNF–BV510 (1:100; BioLegend, 506339)
- anti-TNF–BV5711 (1:100; BioLegend, 506349)
- anti-TNF–PE (1:100; BioLegend, 506306)
- anti-IL-2–Pecy7 (1:100; BioLegend, 503832)
- anti-IL-2–Pacific Blue (1:100; BioLegend, 503820)
- anti-IL-2–BV510 (1:100; BioLegend, 503833)

Samples were analyzed on a BD FACSymphony or a Beckman CytoFLEX.

### In vivo depletion of B cell

Anti-mouse CD19 (200 μg per mouse, BioXCell *In vivo*MAb, #BE0150) was injected (i.p.) once a week for three consecutive weeks (total of three injections) five days before B16F10-OVA inoculation (2.5 × 10^5^ cells; i.d.; hindpaw). Blood samples were taken to confirm depletion. Following the B cell depletion and B16F10-OVA cells inoculation, OPRL1 antagonist (SB612111, 3mM) was daily injected i.d. in five points around the tumor (treatment began one day after the melanoma cells were injected), and tumour growth was measured daily by a handheld digital caliper and evoked thermal hypersensivity was assessed every three days.

### N/OFQ and SB612111 treatment

Orthotopic B16F10-OVA cells (5 × 10^5^ cells, i.d., flank or 2.5 × 10^5^ cells, i.d., hindpaw) were inoculated into male and female C57BL/6 mice. One day later, the mice were treated with either nociceptin recombinant (N/OFQ, 0.6 μg/kg) or OPRL1 blocker (SB612111, 3mM) or vehicle control in five points around the tumor. Thirteen (SB612111) and fourteen (N/OFQ) days after tumor inoculation, the effect of N/OFQ and SB612111 on tumour growth was measured daily by a handheld digital caliper. Mice were euthanized, and tumor-infiltrating cells were immunophenotyped by flow cytometry using a BD FACSymphony or a Beckman CytoFLEX.

### BIBN4096

Starting one day after tumour inoculation, BIBN4096 (Tocris, 4561; 5 mg kg−1)^65^ was administered systemically (i.p., 50 μl) on alternate days to eight-week-old male and female mice.

### Thermal hypersensitivity

To measure thermal sensitivity, the mice were placed on a glass plate of a Hargreaves’s apparatus (Ugo Basile)^66^ and stimulated using radiant heat (infrared beam). The infrared beam intensity was set at 44 and calibrated to result in a withdrawal time of around 12 seconds in acclimatized wild-type mice. An automatic cut-off was set to 25 s to avoid tissue damage. The radiant heat source was applied to the dorsal surface of the hindpaw and latency was measured as the time for the mouse to lift, lick or withdraw the paw^66^.

Before any treatment, the mice were allowed to acclimatize in the apparatus (minimum of three consecutive days: 1 h per session) and three baseline measurements were taken on the following day. In some instances, SB612111 (3mM, 20 μL), or vehicle (20 μL) were injected in the left and right hindpaw, respectively, and thermal hypersensitivity was measured in both hindpaws every other day for 11 days. For the N/OFQ treatment, 0.6 μg/kg of N/OFQ (20 μL) or vehicle (20 μL) were injected in the left and right hindpaw respectively, and thermal hypersensitivity was measured in both hindpaws at 0, 1, 3, and 6 hours.

In other instances, anti-CD19 treatment to deplet B cells was performed, following by the intradermal inoculation of B16F10-OVA cells (2.5 × 10^5^ cells; i.d.) into the mouse’s left hindpaw. One day later, either SB612111 (3mM, 20 μL), or vehicle (20 μL) were injected in the left paw and thermal pain hypersensitivity was measured every three days for 18 days.

### Evoked Mechanical Sensitivity

Mice were acclimated for 30–60 minutes in a temperature- and light-controlled room within individual acrylic chambers (15 × 4 × 4 cm), placed on a stainless-steel mesh platform. Mechanical stimuli were applied to the hindpaw using a series of 8 von Frey monofilaments (Stoelting, Wooddale, IL) of increasing stiffness, each applied with sufficient force to slightly bend the filament. A withdrawal or licking response within 4 seconds was considered positive. The 50% withdrawal threshold was determined using the up-down method, continuing until 4 measurements were recorded after the first change in response direction, as described by Dixon (1980)^67^ and Chaplan et al. (1994)^68^. Data are presented as the calculated 50% mechanical withdrawal threshold.

### Spontaneous Mechanical Sensitivity

Spontaneous pain behavior was assessed using the Blackbox Bio^69^, a bottom-up imaging system that simultaneously captured body pose and paw surface luminance in freely moving mice without applied stimulation. Mice were placed in a black acrylic chamber situated on a borosilicate glass floor illuminated from below with 850-nm near-infrared (NIR) LEDs. A NIR-sensitive camera positioned 30 cm below the glass recorded continuous behavior in the dark at 50 Hz. Alternating video frames captured the animal’s paw-floor contact (via FTIR-based luminance) by toggling the illumination source, controlled by a Raspberry Pi microcomputer.

Paw luminance, serving as a proxy for mechanical pain sensitivity, was quantified from FTIR frames by extracting a fixed pixel region centered on the hind paw positions predicted by a DeepLabCut neural network. Luminance values were scaled within each recording using a min-95 quantile normalization to account for inter-animal variability as described before^69^.

### CGRP release from skin explant

Tumour-surrounding skin was collected using 10-mm punch biopsies from mice treated with SB612111 (3mM) and anti-CD19 (200ug) + SB612111. The biopsies were transferred into 24-well plates and incubated in DMEM containing 1 μl ml−1 of protease inhibitor (Sigma, P1860) and capsaicin (1 μM, Sigma, M2028). After a 30-min incubation (37 °C), the supernatant was collected and the release of CGRP was analysed using a commercial enzyme-linked immunosorbent assay (ELISA)^70^ (Cayman Chemical, 589001).

### N/OFQ and SB612111 -induced B16F10 elimination

A total of 1 × 10^5^ B16F10-mCherry-OVA cells were cultured overnight. One day after, the cells were treated with either N/OFQ (1, 10, 20, and 500 ng/mL) or SB612111 (1, 10, 20, and 100 uM). After three days, the cells were detached by trypsin (Gibco, 2062476) and collected by centrifugation (5 min at 1,300 rpm), stained using anti-Annexin V, 7-AAD (BioLegend, 640930) for 20 min at 4 °C, and immunophenotyped by flow cytometry using a Beckman CytoFLEX.

### Quantitative PCR (qPCR)

Total RNA was isolated from cell pellets (1–1.5×10^6^ cells), cultured B-cells, or whole trigeminal ganglia (TG) using the RNeasy Plus Mini Kit (Qiagen). Reverse transcription was performed with the Quantitect Reverse Transcription Kit (Qiagen) according to the manufacturer’s instructions, and the resulting cDNA was diluted to 5 ng/μL. For RNA isolation and gene expression analysis from fluorescently sorted tongue/tumor-infiltrating leukocytes, the Cells-to-CT 1-Step TaqMan Kit (Thermo Fisher Scientific) was used according to the manufacturer’s protocol. Gene expression was quantified using TaqMan Gene Expression Assays and TaqMan Fast Advanced Master Mix (Thermo Fisher Scientific) on a 96-well QuantStudio 3 Real-Time PCR System (Thermo Fisher Scientific). GAPDH/Gapdh or ACTB/Actb served as internal controls. All samples were run in duplicate or triplicate to minimize pipetting errors. Relative fold changes in gene expression were calculated via the 2−ΔΔ^Ct^ method (e.g., cancer mice vs. sham mice; B-cell treatment vs. vehicle).

### Single-Cell Analysis

For single-cell analysis, DiI-positive single neurons were identified under fluorescence microscopy and collected using a glass capillary (World Precision Instruments) controlled by a micromanipulator (Sutter Instruments). Cell size was not considered for selection. Each neuron was transferred into a 0.2 mL PCR tube containing 9 μL of single-cell lysis buffer with DNase I from the Single Cell-to-CT Kit (Thermo Fisher Scientific). After a 5-minute incubation, samples were immediately stored at −80 °C. Cells were collected within 1 hour of removal from the incubator and within 8 hours of animal euthanasia. Reverse transcription, cDNA pre-amplification, and qPCR were performed following the manufacturer’s guidelines. We used Life Technologies assays for Oprl1 (Mm00440563_m1), with Gusb (Mm01197698_m1) as an internal control. Relative quantification was assessed using the 2−ΔCt method. For single-cell PCR, any cell with a Gusb Ct value ≥28 was excluded. Genes were considered non-expressed if no Ct was detected or if the Ct value exceeded 38.

### Survival analysis of patients with melanoma

OncoLnc (http://www.oncolnc.org/) contains survival data for 8,647 patients from 21 cancer studies performed by TCGA^54^. Using OncoLnc, we assessed the transcript expression of *PNOC* and *OPRL1* genes skin cutaneous carcinoma (SKCM) and head and neck squamous cell carcinoma (HNSCC) tumour biopsies from the TCGA database. Kaplan–Meier curves show the survival of the patients after segregation into two groups defined by their low or high expression of a gene of interest. Details of patients can be found in TCGA^54^ and computational analyses can be found at https://doi.org/10.7717/peerj-cs.67.

### Statistics

Statistical significance was determined using GraphPad Prism (Dotmatics, v.9) and calculated using simple linear regression analysis, Mantel–Cox regression, one-way or two-way ANOVA for multiple comparisons and two-sided unpaired Student’s t-test for single variable comparison. *p* value <0.05 was considered significant.

## Supplementary Material

1
