# Supplementary material for: OPIOID-EXPRESSING B CELLS SILENCE TUMOR-INFILTRATING NOCICEPTOR NEURONS
Source: Res Sq. 2025 Sep 3:rs.3.rs-7389517. Preprint. [Version 1] doi: 10.21203/rs.3.rs-7389517/v1 (PMC12425075; doi:10.21203/rs.3.rs-7389517/v1)
Supplement: 1 [file NIHPPRS7389517V1-supplement-1.pdf]

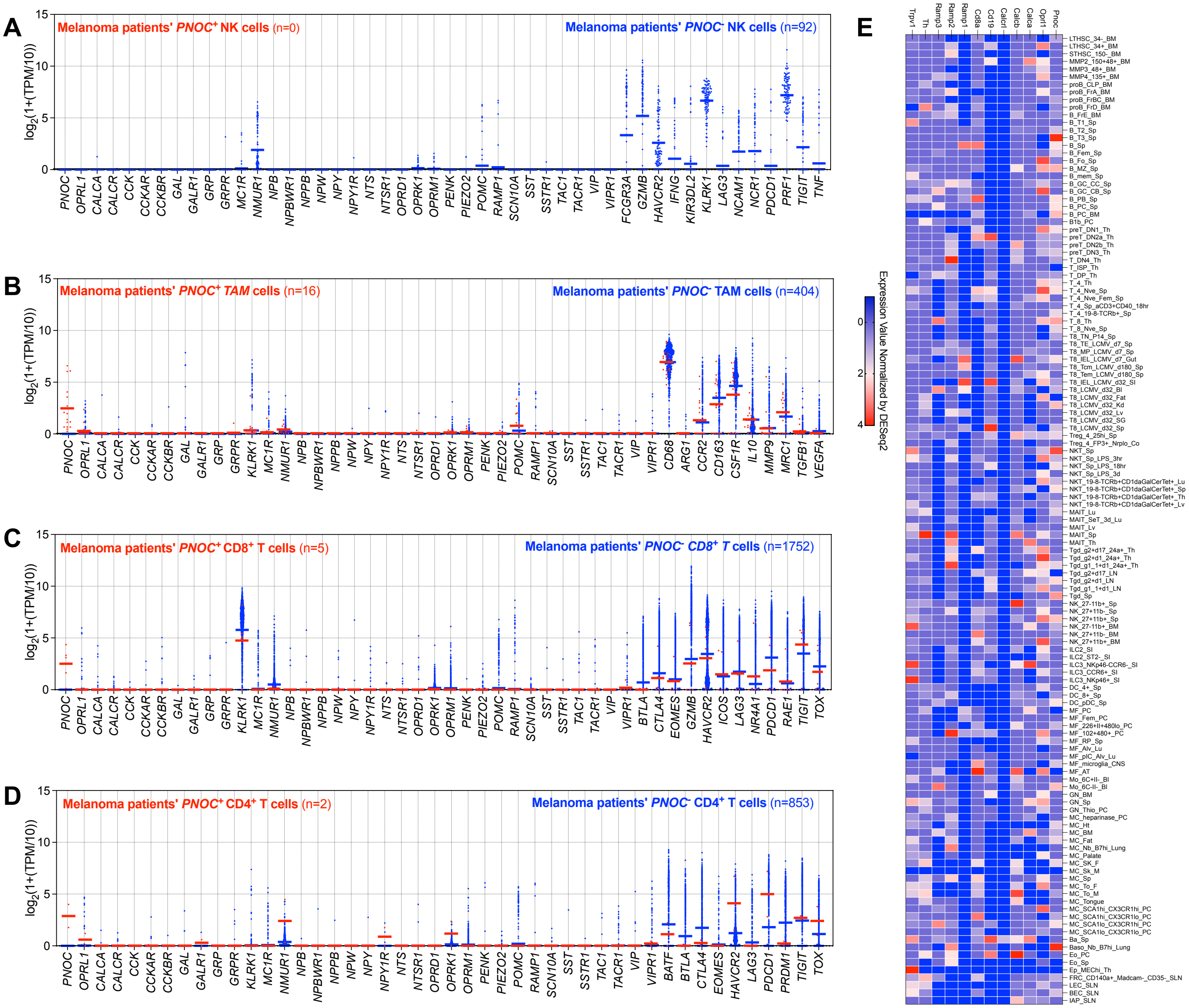
A
Melanoma patients' PNOC+ NK cells (n=0)
Melanoma patients' PNOC- NK cells (n=92)
log2(1+(TPM/10))
PNOC OPRL1 CALCA CALCR CCK CCKAR CCKBR GAL GALR1 GRP GRPR MC1R NMUR1 NPB NPBWR1 NPPB NPW NPY NPY1R NTS NTSR1 OPRD1 OPRK1 OPRM1 PENK PIEZO2 POMC RAMP1 SCN10A SST SSTR1 TAC1 TACR1 VIP VIPR1 FCGR3A GZMB HAVCR2 IFNG KIR3DL2 KLRK1 LAG3 NCAM1 NCR1 PDCD1 PRF1 TIGIT TNF
B
Melanoma patients' PNOC+ TAM cells (n=16)
Melanoma patients' PNOC- TAM cells (n=404)
log2(1+(TPM/10))
PNOC OPRL1 CALCA CALCR CCK CCKAR CCKBR GAL GALR1 GRP GRPR KLRK1 MC1R NMUR1 NPB NPBWR1 NPPB NPW NPY NPY1R NTS NTSR1 OPRD1 OPRK1 OPRM1 PENK PIEZO2 POMC RAMP1 SCN10A SST SSTR1 TAC1 TACR1 VIP VIPR1 CD68 ARG1 CCR2 CD163 CSF1R IL10 MMP9 MRC1 TGFB1 VEGFA
C
Melanoma patients' PNOC+ CD8+ T cells (n=5)
Melanoma patients' PNOC- CD8+ T cells (n=1752)
log2(1+(TPM/10))
PNOC OPRL1 CALCA CALCR CCK CCKAR CCKBR GAL GALR1 GRP GRPR KLRK1 MC1R NMUR1 NPB NPBWR1 NPPB NPW NPY NPY1R NTS NTSR1 OPRD1 OPRM1 PENK PIEZO2 POMC RAMP1 SCN10A SST SSTR1 TAC1 TACR1 VIP VIPR1 BTLA CTLA4 EOMES GZMB HAVCR2 ICOS LAG3 NR4A1 PDCD1 RAE1 TIGIT TOX
D
Melanoma patients' PNOC+ CD4+ T cells (n=2)
Melanoma patients' PNOC- CD4+ T cells (n=853)
log2(1+(TPM/10))
PNOC OPRL1 CALCA CALCR CCK CCKAR CCKBR GAL GALR1 GRP GRPR KLRK1 MC1R NMUR1 NPB NPBWR1 NPPB NPW NPY NPY1R NTS NTSR1 OPRD1 OPRK1 OPRM1 PENK PIEZO2 POMC RAMP1 SCN10A SST SSTR1 TAC1 TACR1 VIP VIPR1 BATF BTLA CTLA4 EOMES HAVCR2 LAG3 PDCD1 PRDM1 TIGIT TOX
E
Trpv1 Th Ramp3 Ramp2 Ramp1 Cd8a Cd19 Calcrl Calcb Calca Oprl1 Pnoc
Expression Value Normalized by DESeq2

Supplementary Fig. 1. *PNOC* expression is restricted to tumor-infiltrating B-cells.

A-D, *In silico* analysis of single-cell RNA sequencing data from tumor-infiltrating lymphocytes in human melanoma showed no *PNOC*-expressing NK cells out of 92 total NK cells (**A**), 18 *PNOC*-expressing TAMs out of 404 total TAMs (**B**), 5 *PNOC*-expressing $CD8^+$ T-cells out of 1,752 total $CD8^+$ T-cells (**C**), and 2 *PNOC*-expressing $CD4^+$ T-cells out of 853 total $CD4^+$ T-cells (**D**).

E, ImmGen RNA sequencing of various leukocyte subpopulations[58] indicates limited *PNOC* expression across cell types, with notable expression confined to B-cells and a subpopulation of splenic NK^+ T-cells.

*Data are shown as violin plots presenting mRNA expression data as $\log_2(1 + (TPM/10))$ on a logarithmic scale (**A-D**) or as heatmap presenting DESeq2-normalized data on a logarithmic scale (**E**). n as follows: **A:** 0 ($PNOC^{pos}$) and 92 ($PNOC^{neg}$); **B:** 16 ($PNOC^{pos}$) and 404 ($PNOC^{neg}$); **C:** 5 ($PNOC^{pos}$) and 1752 ($PNOC^{neg}$); **D:** 2 ($PNOC^{pos}$) and 853 ($PNOC^{neg}$); **E:** 127.*

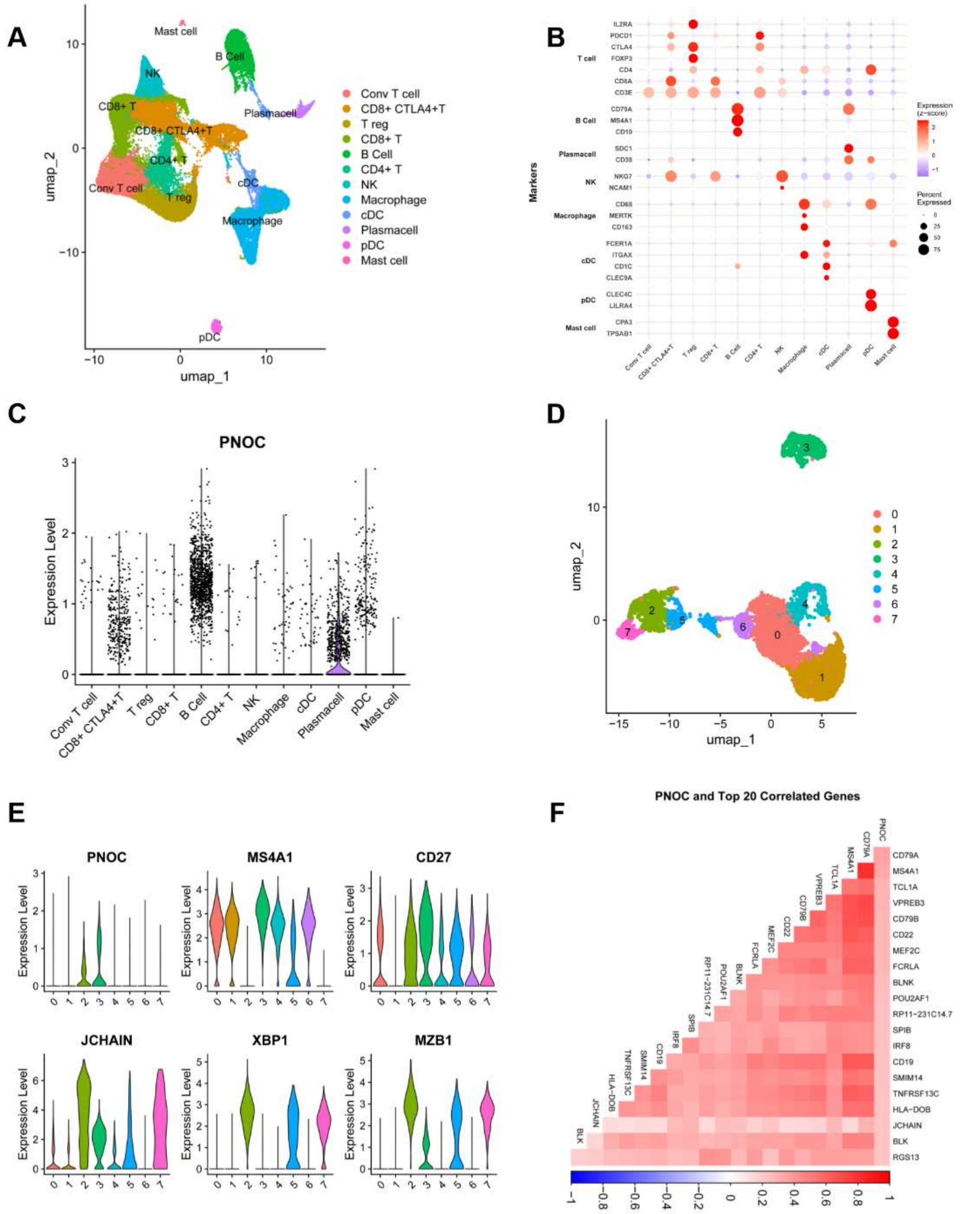

Supplementary Fig. 2. *PNOC* expression is restricted to tumor-infiltrating B cells in patients with head and neck cancer.

A-F, *In silico* analysis of single-cell RNA sequencing data from tumor-infiltrating leukocytes in head and neck squamous cell carcinoma (HNSCC) patient biopsies. UMAP plot (**A**) and annotation dot plot (**B**) show various immune cell populations. A violin plot showing the expression level of *PNOC* across the identified immune cell clusters (**C**). UMAP plot displays the sub-clustering of B cells and plasma cells (**D**). Expression distributions of *PNOC* and key B cell/plasma cell-related genes across sub-clusters (**E**). Heatmap illustrating the Pearson correlation coefficients between *PNOC* and its top 20 most correlated genes in the HNSCC (**F**).

*Data are shown as dimension-reduction visualizations (UMAP) for single-cell data, with each color/cluster representing different cell types or subpopulations within tumor-infiltrating leukocytes (**A**), and tumor-infiltrating B-cells and plasma cells (**D**); as violin plots illustrating the distribution of normalized gene expression levels across immune cell populations (**C**) or specific B-cell and plasma cell sub-clusters (**E**); or as heatmaps comparing gene expression correlations (**F**).*

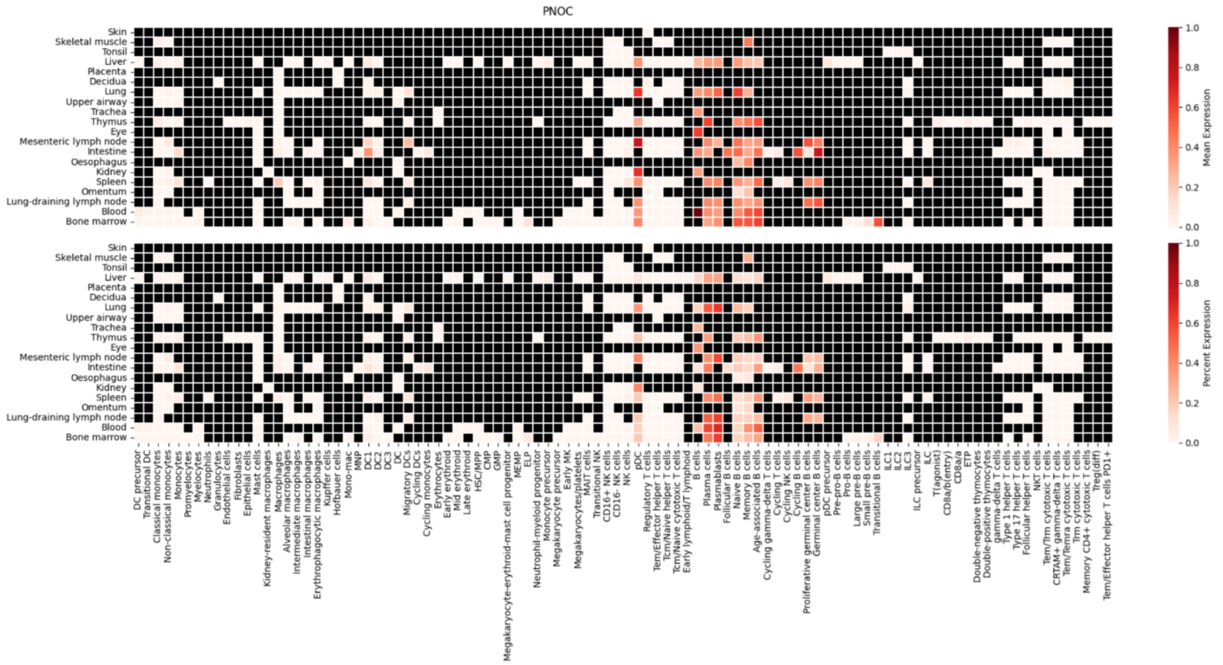

Supplementary Fig. 3. ***PNOC* expression across various immune cell types and tissues.**

In silico analysis of *PNOC* expression levels using the CellTypist Encyclopedia (v2) database for human immune cells[59,60]. The dataset reveals higher *PNOC* expression in pDC, plasma cells, and different subtypes of B cells.

Data are shown as heatmap.

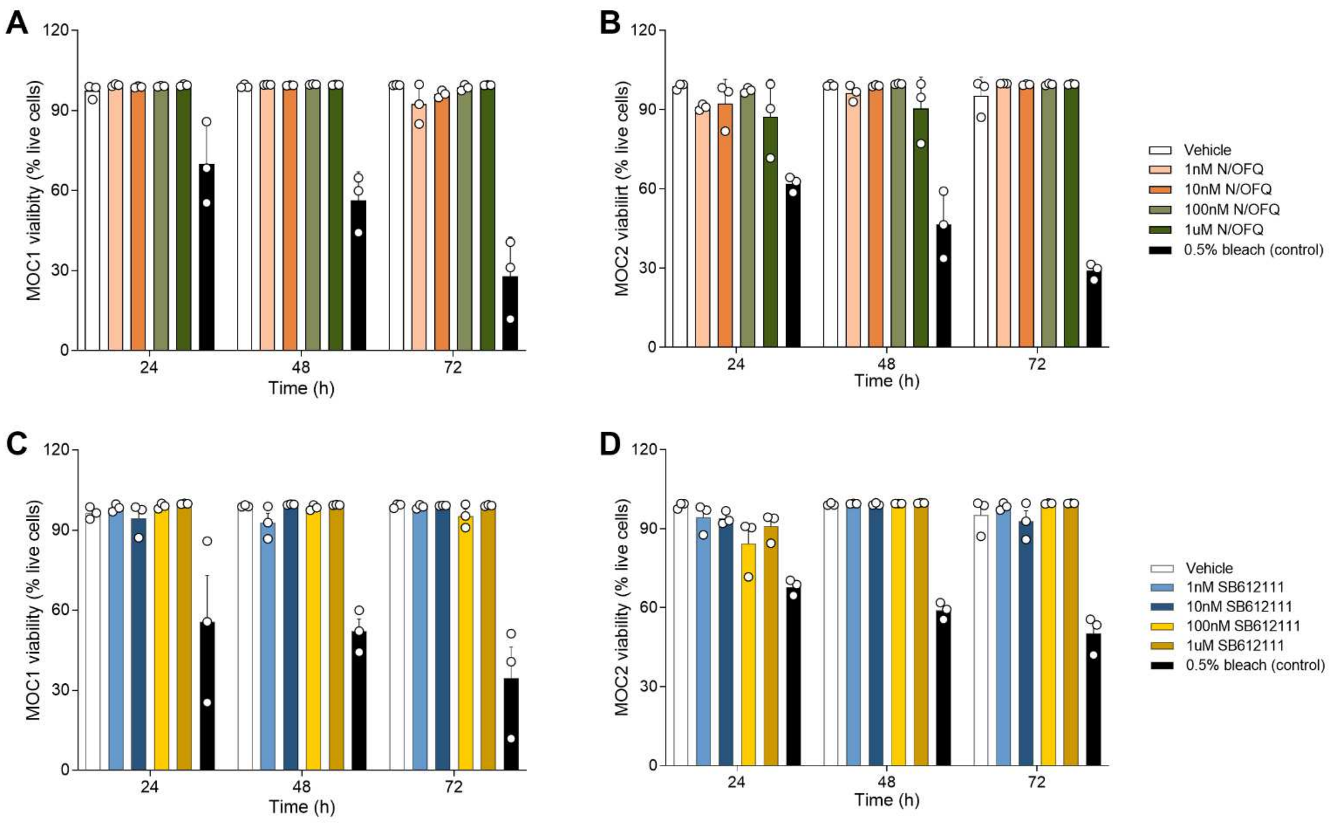

Supplementary Fig. 4. N/OFQ and OPRL1 antagonist do not affect the viability of head and neck squamous cell carcinoma cells.

A-D, MOC1 and MOC2 cells were cultured for 24 hours. In the presence of protease inhibitors, cells were then stimulated with different concentrations of PNOC and the OPRL1 antagonist SB612111 for 24, 48, and 72 hours. Neither PNOC (**A, B**) nor the OPRL1 antagonist SB612111 (**C, D**) altered the cell viability.

*Data are presented as mean ± s.e.m. (**A-D**). n as follows: 3 per group. p values were determined by nested one-way ANOVA with Bonferroni post hoc test (**A- D**).*

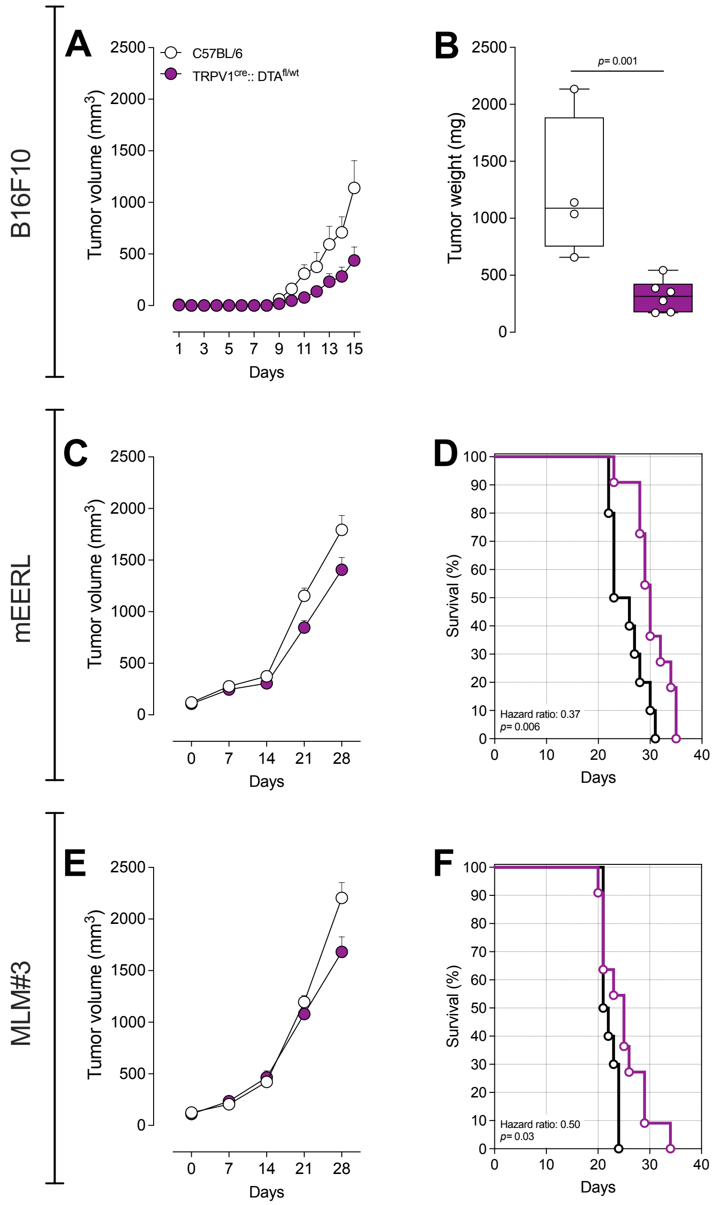

Supplementary Fig. 5. Genetic ablation of nociceptors reduces tumor growth.

A, B, Orthotopic B16F10 cells (1×10^5 cells, i.d.) were inoculated into the flank of male mice with sensory neurons intact (C57BL/6) or ablated ($TRPV1^{cre}$::$DTA^{fl/wt}$). Fifteen days after tumour inoculation, sensory-neuron-ablated mice have reduced tumour growth (**A**), and tumor weight (**B**).

C, D, Orthotopic mEERL cells (1×10^5 cells, i.d.) were inoculated into the flank of male mice with sensory neurons intact (C57BL/6) or ablated ($TRPV1^{cre}$::$DTA^{fl/wt}$). Twenty-eight days after tumour inoculation, sensory-neuron-ablated mice have reduced tumour growth (**C**), and increased survival (**D**).

E, F, Orthotopic MLM#3 cells (1×10^5 cells, i.d.) were inoculated into the flank of male mice with sensory neurons intact (C57BL/6) or ablated ($TRPV1^{cre}$::$DTA^{fl/wt}$). Fifteen days after tumour inoculation, sensory-neuron-ablated mice have reduced tumour growth (**E**), and increased survival (**F**).

*Data are presented as mean ± s.e.m. (**A, C, E**), as box-and-whisker plots (extending from the minimum to the maximum values, with the box spanning the 25th to 75th percentiles and the middle line indicating the median), where individual data points are shown (**B**), and as Mantel–Cox regression (**D, F**). n as follows: **A, B:** 4 (C57BL/6) and 6 ($TRPV1^{cre}$::$DTA^{fl/wt}$); **C-F:** 10 (C57BL/6) and 11 ($TRPV1^{cre}$::$DTA^{fl/wt}$). p values were determined by two-way ANOVA with Bonferroni post hoc test (**A, C, E**), two-sided unpaired Student's t-test (**B**), or Mantel–Cox regression (**D, F**).*

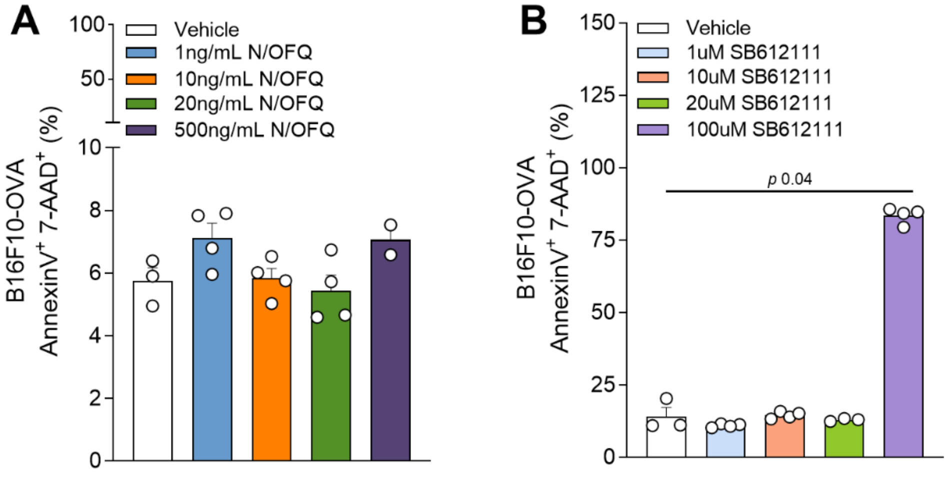

Supplementary Fig. 6. N/OFQ and OPRL1 antagonist do not affect the viability of melanoma cells.

A-B, B16F10-OVA cells were cultured for 24 h with a protease inhibitor. Different concentrations of N/OFQ (**A**) and the OPRL1 antagonist SB612111 (**B**) were added to the cultures for an additional 24 h. Neither N/OFQ (**A**) nor SB612111 (**B**) affected cell viability.

*Data are shown as mean ± s.e.m (**A, B**). n as follows: 3-4 per group. p values were determined by nested one-way ANOVA with Bonferroni post hoc test (**A, B**).*

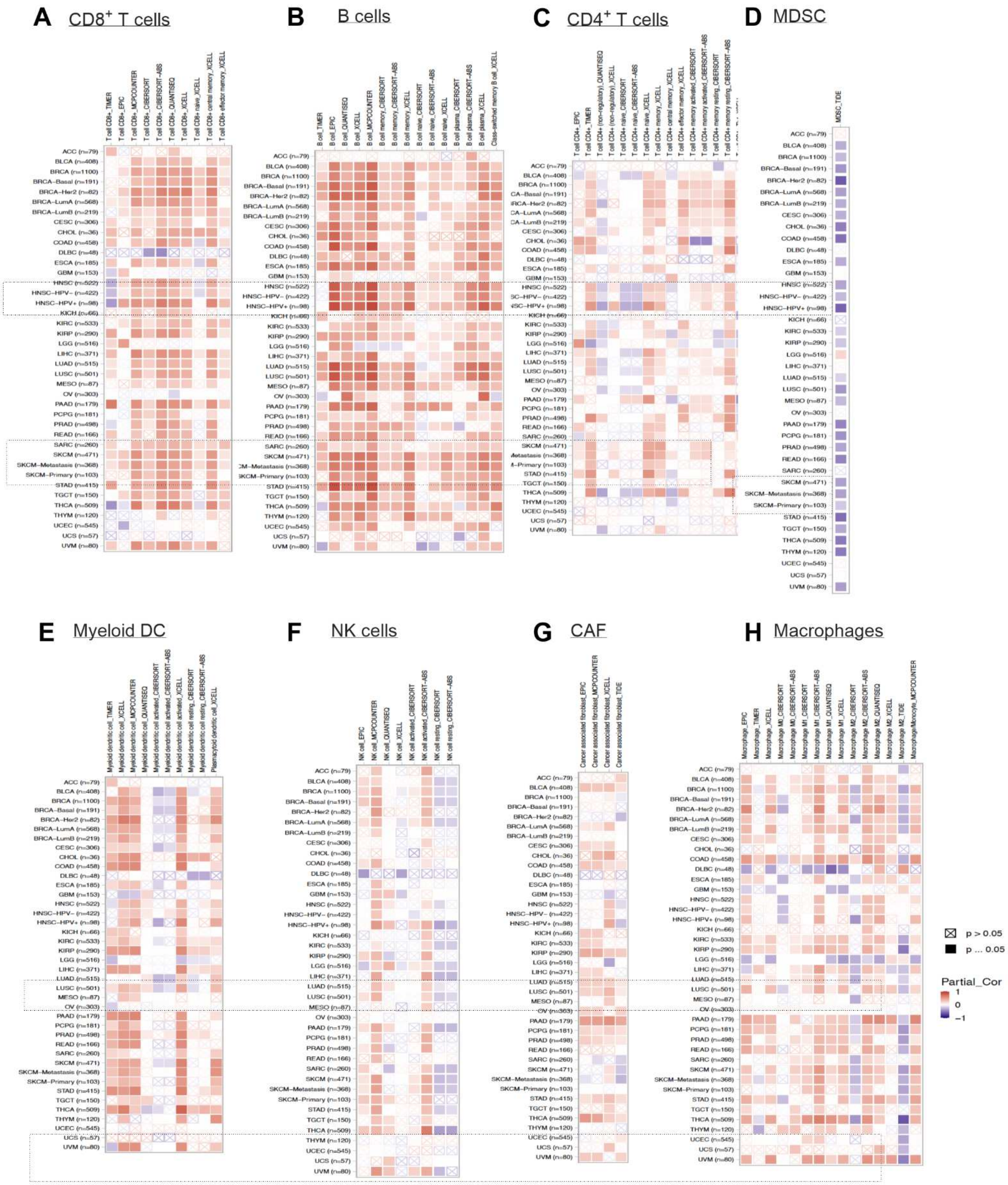
A CD8+ T cells
B B cells
C CD4+ T cells
D MDSC
E Myeloid DC
F NK cells
G CAF
H Macrophages
p > 0.05
p ... 0.05
Partial_Cor
1
0
−1

Supplementary Fig. 7. ***PNOC* expression correlates with anti-tumor immunity across cancer types.**

A-I, *In silico* analysis of immune infiltrates and *PNOC* expression in TCGA cancer types. Heatmap visualizes the partial Spearman's correlation coefficients (rho) between *PNOC* expression and immune cell types and infiltration levels in TCGA cancer types. Immune cell infiltration was estimated using transcriptomic deconvolution methods (determined by bulk RNA sequencing of tumors). In skin cutaneous melanoma (SKCM) and head and neck squamous cell carcinoma (HNSCC), high *PNOC* expression positively correlates with $CD8^{+}$ T cell infiltration (**A**), B-cell infiltration (**B**), and myeloid dendritic cell infiltration (**E**); while showing a negative correlation with myeloid-derived suppressor cell (MDSC) infiltration (**C**). However, *PNOC* expression exhibits only a limited correlation with $CD4^{+}$ T-cells (**D**), NK cells (**F**), cancer-associated fibroblasts (CAF) (**G**), and macrophages (**H**).

*Data are shown as heatmap indicating partial Spearman's correlation coefficient (rho) between PNOC expression and immune cell types of infiltration levels in TCGA cancer types (**A-I**). n for each cancer type is displayed on the plots.*

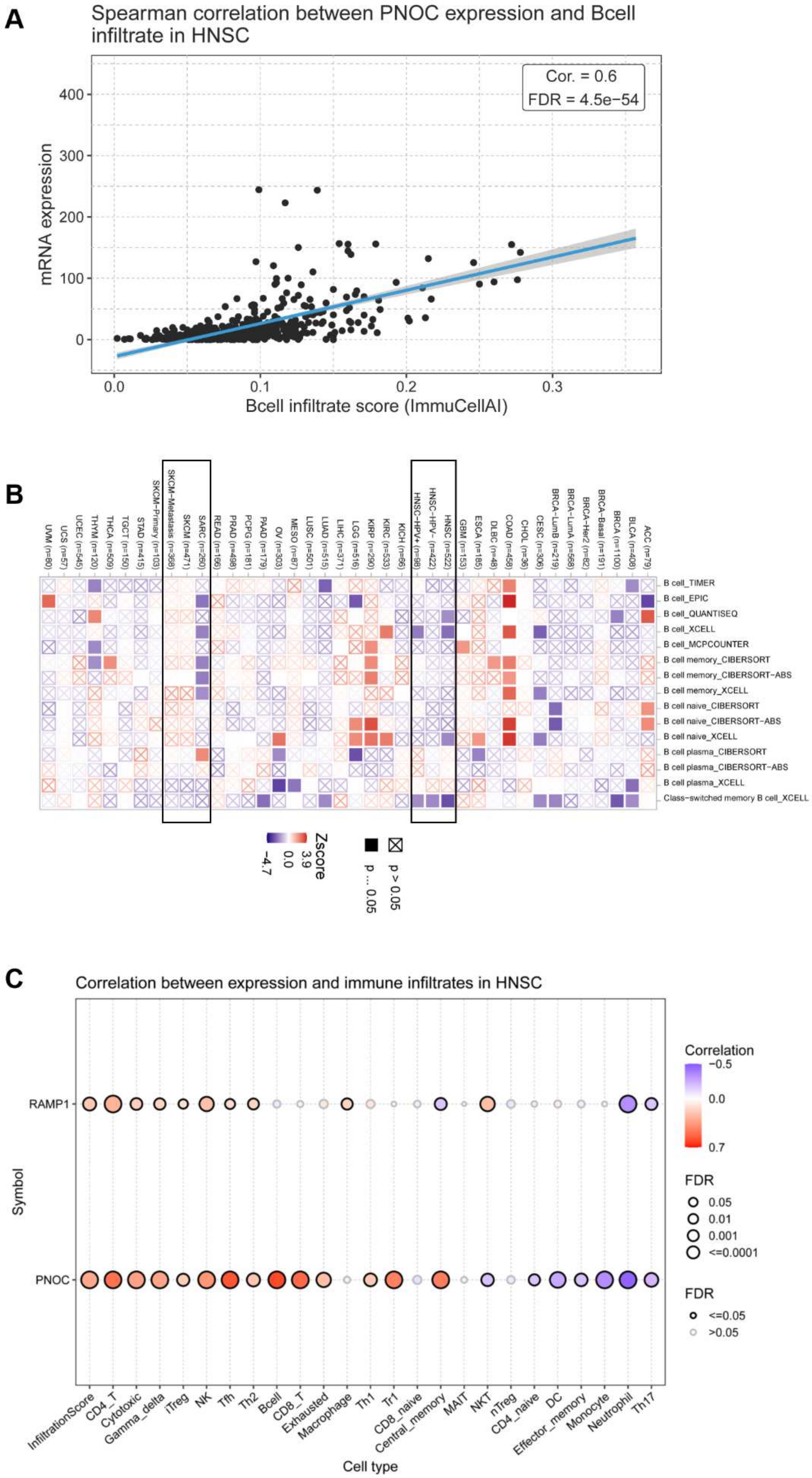
A
Spearman correlation between PNOC expression and Bcell infiltrate in HNSC
Cor. = 0.6
FDR = 4.5e−54
mRNA expression
0
100
200
300
400
0.0
0.1
0.2
0.3
Bcell infiltrate score (ImmuCellAI)
B
B cell_TIMER
B cell_EPIC
B cell_QUANTISEQ
B cell_XCELL
B cell_MCPCOUNTER
B cell memory_CIBERSORT
B cell memory_CIBERSORT-ABS
B cell memory_XCELL
B cell naive_CIBERSORT
B cell naive_CIBERSORT-ABS
B cell naive_XCELL
B cell plasma_CIBERSORT
B cell plasma_CIBERSORT-ABS
B cell plasma_XCELL
Class-switched memory B cell_XCELL
Zscore
3.9
0.0
−4.7
p > 0.05
C
Correlation between expression and immune infiltrates in HNSC
RAMP1
PNOC
Symbol
Correlation
−0.5
0.0
0.7
FDR
0.05
0.01
0.001
<=0.0001
FDR
<=0.05
>0.05
InfiltrationScore
CD4_T
Cytotoxic
Gamma_delta
iTreg
NK
Tfh
Th2
Bcell
CD8_T
Exhausted
Macrophage
Th1
Tr1
CD8_naive
Central_memory
MAIT
NKT
nTreg
CD4_naive
DC
Effector_memory
Monocyte
Neutrophil
Th17
Cell type

Supplementary Fig. 8. ***PNOC* expression correlates with B-cell infiltration**.

A, *In silico* analysis of head and neck squamous cell carcinoma (HNSCC) patient biopsies (bulk RNA sequencing)[61] revealed a positive correlation between the B-cell infiltration score (calculated using ImmuCellAI) and *PNOC* expression.

B, *In silico* analysis of association between B cell infiltration and survival across TCGA cancer types, adjusted for *PNOC* expression. Infiltration levels of B cell subsets were estimated using multiple deconvolution methods. B-cell infiltration score, derived from multiple deconvolution methods matrices provides a surrogate measure of B-cell presence in tumors based on bulk RNA sequencing. Across multiple cancer types, *PNOC* expression positively correlated with the B-cell gene signature, especially in HNSCC.

C, *In silico* analysis of correlation between *RAMP1/PNOC* expression and immune infiltrates in HNSCC using TCGA data. The color of each circle indicates the Pearson correlation coefficient. The size of the circles represents the FDR, indicating the statistical significance of the correlation. The relationship between *PNOC* and *RAMP1* gene expression and various immune cell populations using multiple gene signature profiles are displayed on the plot. In HNSCC, high *PNOC* expression positively correlate with both B- and T-cell infiltration.

*Data are shown as scatter plot with a linear regression line **A**), as heatmap (**B**), or as correlation dot plot (**C**). n as follows: **A, C:** 522; **B:** displayed on the plot for each cancer type.*

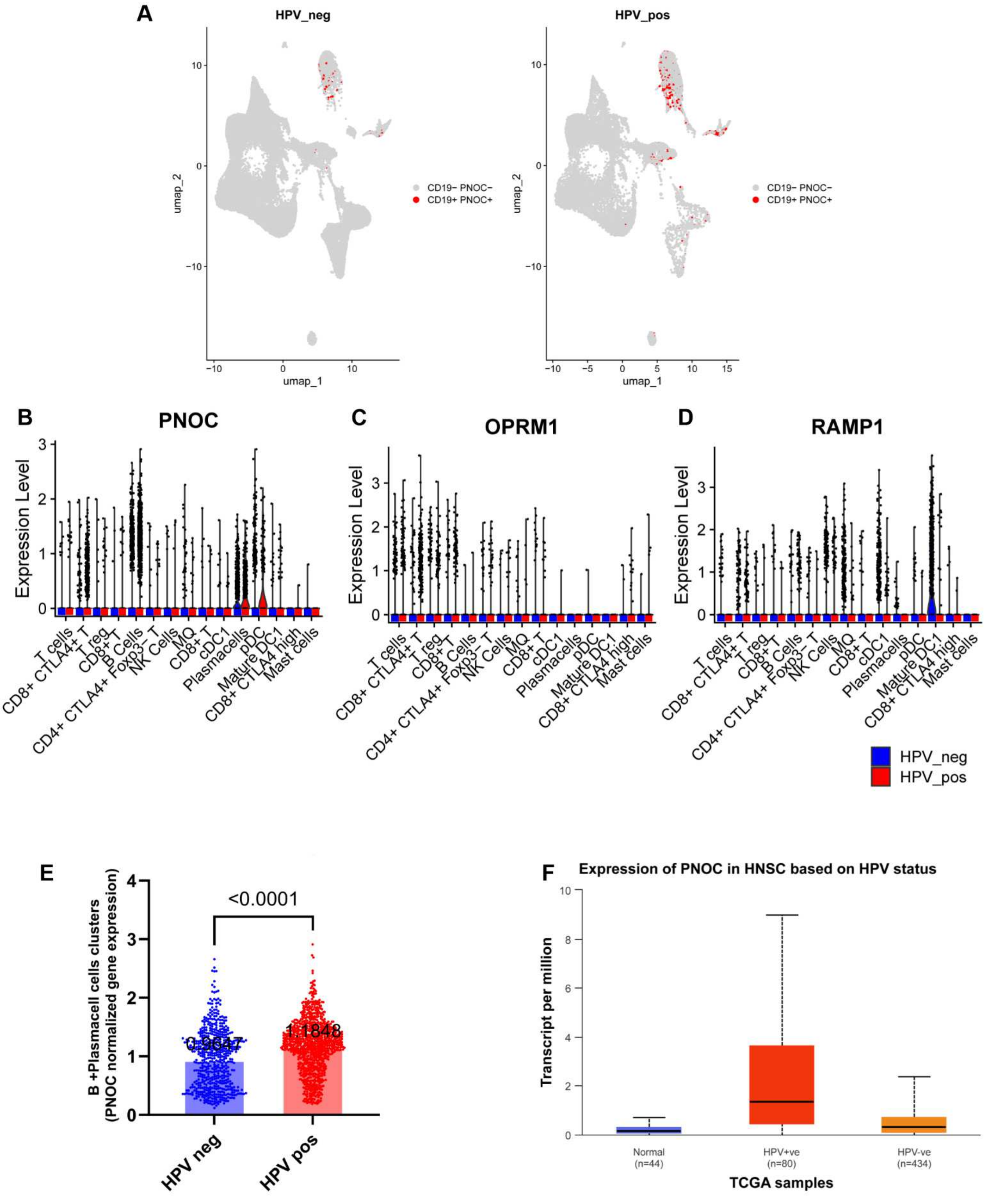

A
HPV_neg
HPV_pos
umap_1
umap_2
CD19− PNOC−
CD19+ PNOC+
B
PNOC
C
OPRM1
D
RAMP1
Expression Level
T cells
CD8+ CTLA4+ T
T reg
CD8+ T
B Cells
CD4+ CTLA4+ Foxp3− T
NK Cells
MQ
cDC1
Plasmacells
pDC
Mature DC1
CD8+ CTLA4 high
Mast cells
HPV_neg
HPV_pos
E
B +Plasmacell cells clusters (PNOC normalized gene expression)
<0.0001
0.9047
1.1848
HPV neg
HPV pos
F
Expression of PNOC in HNSC based on HPV status
Transcript per million
Normal (n=44)
HPV+ve (n=80)
HPV-ve (n=434)
TCGA samples

Supplementary Fig. 9. ***PNOC* is overexpressed in HPV^+ head and neck cancer patient–infiltrating B-cells.**

A-F, *In silico* analysis of single cell RNA-sequencing data from head and neck squamous cell carcinoma (HNSCC) patient biopsies (GSE139324) —including samples from seven HPV-positive and nineteen HPV-negative patients. The data reveals distinct expression profiles for $CD19^+PNOC^+$ B-cells in HPV^- and HPV^+ patients. UMAP plots illustrate this distribution, while differential expression analyses for *PNOC* (**B**), *OPRM1* (**C**), and *RAMP1* (**D**) indicated that, within plasma B-cell populations, *PNOC* is notably overexpressed in HPV^+ patients, who are known to experience less cancer-related pain than their HPV^- counterparts[25]. **F,** *In silico* analysis of TCGA data indicates that HPV^+ HNSCC patients exhibit higher *PNOC* expression relative to HPV^- patients and healthy controls. Data were obtained using the UALCAN web tool[62,63].

*Data are presented as dimension-reduction visualizations (UMAP) (**A**) where each point in the UMAP space represents a single cell, as dot plots showing normalized mRNA counts on a logarithmic scale (**B-E**), or as whisker plots showing TPM expression levels (**F**). n as follows: **E:** 19 (HPV^{neg}) and 7 (HPV^{pos}); **F:** displayed on the plot for each cancer type. p values were determined by two-sided unpaired Student's t-test (**E**) or one-way ANOVA with Bonferroni post hoc (**F**).*

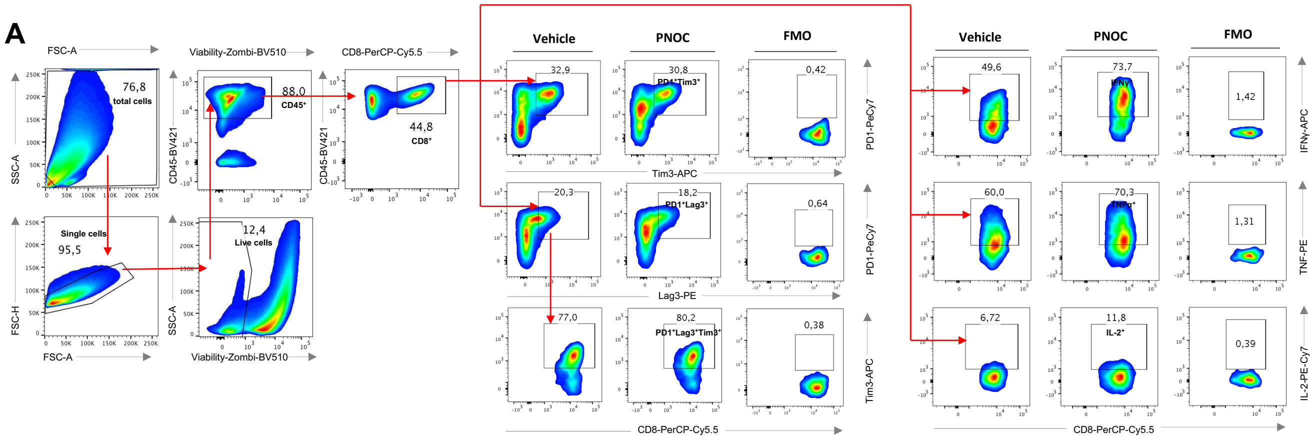

Supplementary Fig. 10. Immunophenotyping analysis of melanoma-infiltrating immune cells.

A, Orthotopic B16F10-OVA cells ($5×10^5$) were inoculated intradermally into the flank of wild-type mice. Starting one day later, mice received daily intradermal injections of either vehicle (50 µL) or recombinant N/OFQ (0.6 µg/kg; 50 µL) at five sites surrounding the tumor. Fourteen days after tumor inoculation, tumors were harvested and analyzed by flow cytometry. Among tumor-infiltrating immune cells, mice treated with N/OFQ exhibited a higher proportion of IFNγ$^+$, TNFα$^+$, and IL-2$^+$ producing CD8$^+$ T-cells compared to vehicle-treated controls.

*Data are shown as representative flow cytometry panels (**A**).*

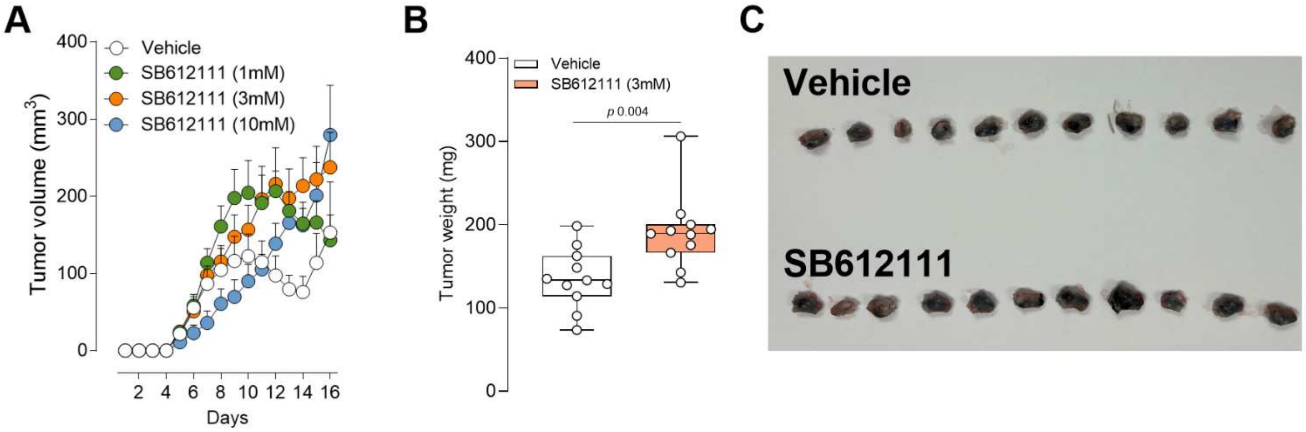

Supplementary Fig. 11. **OPRL1 blockade increased tumor growth.**

A, Orthotopic B16F10-OVA cells (5×10^5) were inoculated intradermally into the flank of eight-week-old wild-type mice. Starting one day later, mice received daily intradermal injections of either vehicle (200 µL) or the OPRL1 receptor antagonist SB612111 (1, 3, and 10 mM; 200 µL; administered at five points around the tumor). Mice treated with 3 mM or 10 mM SB612111 exhibited increased tumor volume compared with vehicle-treated controls.

B, C, Orthotopic B16F10-OVA cells (2×10^5) were inoculated intradermally into the right hind paw of eight-week-old wild-type mice. Starting one day later, mice received daily intradermal injections of either vehicle (200 µL) or SB612111 (3 mM; 200 µL) at five sites surrounding the tumor. Under these conditions, SB612111 increased tumor weight (**B, C**).

*Data are shown as mean ± s.e.m. in (**A**), as box-and-whisker plots with individual data points indicated (**B**), or as a representative image of tumor size (**C**). n as follows: **A:**) 5 (vehicle), 7 (1 mM SB612111), 9 (3 mM SB612111), and 7 (10 mM SB612111); **B, C**: 11 per group. p values were determined by two-way ANOVA with Bonferroni post hoc (**A**) or two-sided unpaired Student's t-test (**B**).*

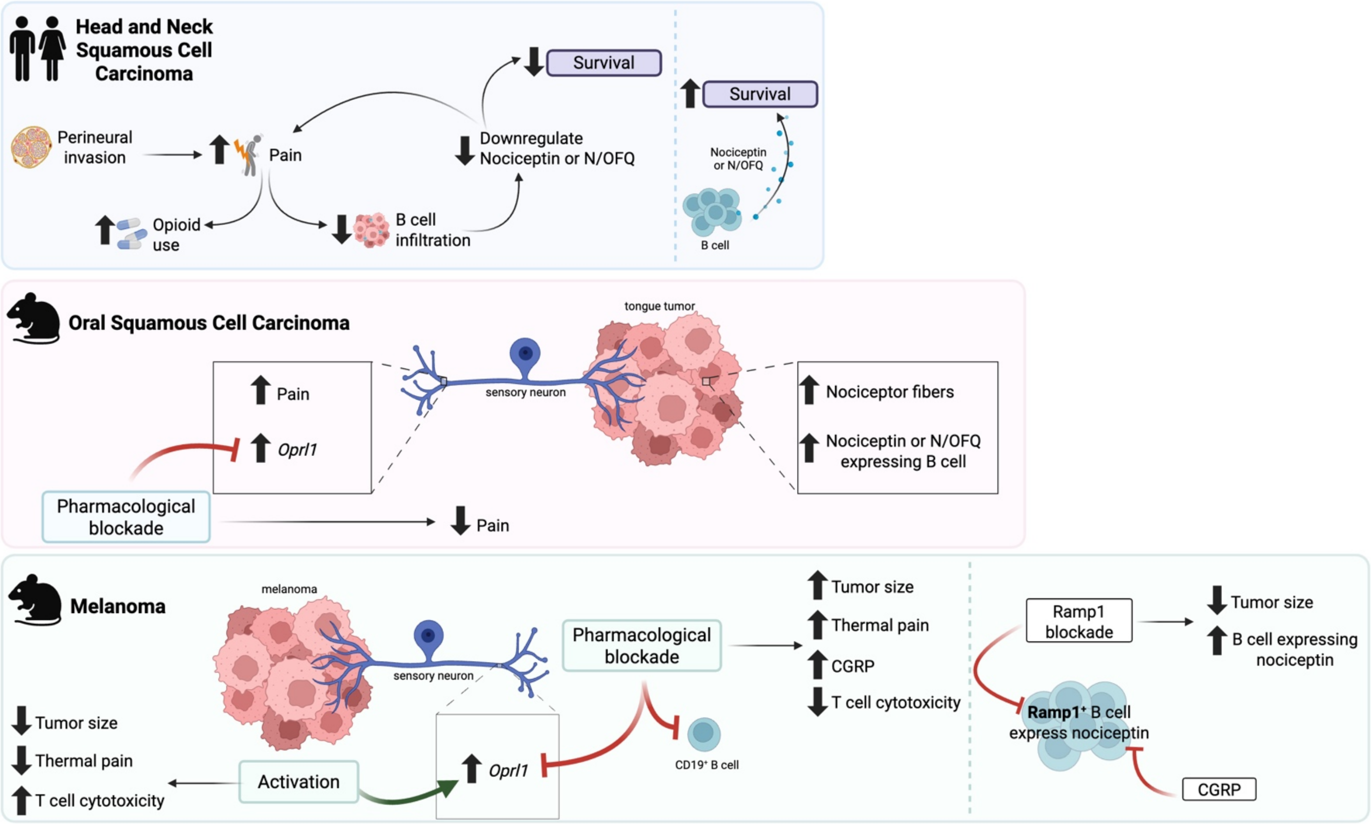

Supplementary Fig. 12. **Role of B cell–derived N/OFQ in modulating pain, immunosurveillance, and tumor progression in different cancer types.**

A, In human head and neck squamous cell carcinoma (HNSCC), perineural invasion leads to cancer pain and consequent higher opioid consumption, correlating with reduced B cell infiltration and downregulation of nociceptin/orphanin (N/OFQ) levels. This immunosuppressive axis worsens survival outcomes. Conversely, restoring B cell-derived N/OFQ improves survival.

B, In mouse oral squamous cell carcinoma (oSCC), tongue tumors are highly innervated by nociceptor fibers and N/OFQ-expressing B cells, amplifying pain and *Oprl1* expression in sensory neurons. Pharmacologically blocking OPRL1 reduces pain.

C, In mouse melanoma, tumors are innervated by sensory neurons that overexpress *Oprl1*, reducing tumor size and thermal pain, and enhancing anti-tumor T cell activity. Pharmacological OPRL1 blockade reverses these effects, promoting tumor growth, thermal pain, and CGRP production while impairing T cell cytotoxicity. Additionally, Ramp1^{+} B cells produce N/OFQ, further driving tumor progression via CGRP signaling. Blocking Ramp1+ diminishes tumor size and increases B cell-derived N/OFQ.
